# Causal relationship between obesity and serum testosterone status in men: A bi-directional mendelian randomization analysis

**DOI:** 10.1371/journal.pone.0176277

**Published:** 2017-04-27

**Authors:** Joel Eriksson, Robin Haring, Niels Grarup, Liesbeth Vandenput, Henri Wallaschofski, Erik Lorentzen, Torben Hansen, Dan Mellström, Oluf Pedersen, Matthias Nauck, Mattias Lorentzon, Lise Lotte Nystrup Husemoen, Henry Völzke, Magnus Karlsson, Sebastian E. Baumeister, Allan Linneberg, Claes Ohlsson

**Affiliations:** 1 Centre for Bone and Arthritis Research, Institute of Medicine, Sahlgrenska Academy, University of Gothenburg, Gothenburg, Sweden; 2 University Medicine Greifswald, Institute of Clinical Chemistry and Laboratory Medicine, Greifswald, Germany; 3 School of Public Health and Preventive Medicine, Monash University, Melbourne, Australia; 4 The Novo Nordisk Foundation Center for Basic Metabolic Research, Faculty of Health and Medical Sciences, University of Copenhagen, Copenhagen, Denmark; 5 Bioinformatics Core Facility, Sahlgrenska Academy, University of Gothenburg, Gothenburg, Sweden; 6 Geriatric Medicine, Department of Internal Medicine and Clinical Nutrition, Institute of Medicine, University of Gothenburg, Gothenburg, Sweden; 7 Research Centre for Prevention and Health, the Capital Region, Copenhagen, Denmark; 8 University Medicine Greifswald, Institute for Community Medicine, Greifswald, Germany; 9 Clinical and Molecular Osteoporosis Research Unit, Department of Orthopaedics and Clinical Sciences, Lund University, Skåne University Hospital (SUS), Malmö, Sweden; 10 Department of Epidemiology and Preventive Medicine, University of Regensburg, Regensburg, Germany; 11 Department of Clinical Experimental Research, Rigshospitalet, Glostrup, Denmark; 12 Department of Clinical Medicine, Faculty of Health and Medical Sciences, University of Copenhagen, Copenhagen, Denmark; Shanghai Diabetes Institute, CHINA

## Abstract

**Context:**

Obesity in men is associated with low serum testosterone and both are associated with several diseases and increased mortality.

**Objectives:**

Examine the direction and causality of the relationship between body mass index (BMI) and serum testosterone.

**Design:**

Bi-directional Mendelian randomization (MR) analysis on prospective cohorts.

**Setting:**

Five cohorts from Denmark, Germany and Sweden (Inter99, SHIP, SHIP Trend, GOOD and MrOS Sweden).

**Participants:**

7446 Caucasian men, genotyped for 97 BMI-associated SNPs and three testosterone-associated SNPs.

**Main outcome measures:**

BMI and serum testosterone adjusted for age, smoking, time of blood sampling and site.

**Results:**

1 SD genetically instrumented increase in BMI was associated with a 0.25 SD decrease in serum testosterone (IV ratio: -0.25, 95% CI: -0.42–-0.09, p = 2.8*10^−3^). For a body weight reduction altering the BMI from 30 to 25 kg/m^2^, the effect would equal a 13% increase in serum testosterone. No association was seen for genetically instrumented testosterone with BMI, a finding that was confirmed using large-scale data from the GIANT consortium (n = 104349).

**Conclusions:**

Our results suggest that there is a causal effect of BMI on serum testosterone in men. Population level interventions to reduce BMI are expected to increase serum testosterone in men.

## Introduction

Observational studies demonstrate that obesity is associated with low serum testosterone (T) [1], but the direction and causality of this relationship is unclear. Most randomized, placebo-controlled trials have indicated that T treatment increases lean mass and reduces fat mass in men with low serum T [2–7], but the overall effect on body weight and BMI in men with different T-status is inconsistent. One possible explanation for the conflicting results regarding the effects of T treatment on body weight and BMI might be that the observational association between low T and high BMI is subject to reverse causation [8–10]. Based on the inverse association between T and obesity-related diseases [11–13], it has been hypothesized that T supplementation could be used as a means to reduce the risk of developing obesity-associated cardio-metabolic diseases in men with low serum T. However, safety concerns have been raised, since some studies have reported an increase in cardiovascular events after T supplementation [14, 15] and randomized controlled trials are still needed [16].

A Mendelian randomization (MR) approach uses genetic variants that index the exposure of interest to test for a causal relationship between exposure and outcome. Since genes can be thought of as randomized and fixed at conception, confounding factors will be equally distributed among different genotypes. As a consequence, MR analyses will be less prone to confounding than the directly observed association. Furthermore, it will be free of reverse causation since a phenotypic trait cannot cause genetic variation.

The most recent and largest genome wide association study (GWAS) on BMI identified 97 independent genetic variants associated with BMI while the largest GWAS on serum T identified three independent genetic variants associated with serum T in men [17, 18].

As the prescription of T to men has increased substantially [19] and its impact on obesity and obesity-related diseases is unclear, we believe it is important to determine the direction and causality of the relatively strong observational relationship between BMI and serum T. The aim of the present study was to identify a possible causative relationship, and its direction of effect, between BMI and serum T using genetic variants as instruments in bi-directional MR analyses.

## Methods

### Participants

This study included participants from five different cohorts of Caucasian men (Inter99, SHIP, SHIP Trend, GOOD and MrOS Sweden) from Denmark, Germany and Sweden. A total of 7446 participants with available genotype and phenotype data were included (Table 1).

**Table 1:** Characteristics of the cohorts.

	GOOD	MrOS Sweden	SHIP	SHIP Trend	INTER99
Characteristics	(n = 929)	(n = 1682)	(n = 1912)	(n = 427)	(n = 2496)
					
Outcomes					
Testosterone—ng/ml	4.7 (1.5)	4.5 (1.7)	4.8 (1.7)	4.0 (1.3)	4.4 (1.6)
BMI—kg/m^2^	22.4 (3.2)	26.3 (3.6)	27.7 (4.0)	27.8 (3.7)	26.8 (4.0)
SHBG—nmol/l	20.4 (7.2)	46.0 (22.9)	51.3 (25.6)	38.3 (14.2)	31.8 (13.1)
					
Covariates					
Age—years	18.9 (0.6)	75.4 (3.2)	50.8 (16.4)	50.1 (14.2)	46.7 (7.9)
Smoking—percent	9%	9%	34%	22%	36%
					
Genetic Risk scores					
_w_GRS_BMI_	88.8 (6.3)	89.0 (6.3)	89.1 (6.2)	88.8 (6.2)	87.6 (6.1)*
_uw_GRS_BMI_	91.4 (6.4)	91.7 (6.3)	91.6 (6.1)	91.5 (6.2)	90.6 (6.0)*
_w_GRS_T_	1.4 (0.6)	1.4 (0.6)	1.4 (0.5)	1.4 (0.5)	1.3 (0.5)
_uw_GRS_T_	2.3 (0.7)	2.4 (0.8)	2.3 (0.7)	2.3 (0.7)	2.3 (0.8)

Values are given as mean with standard deviation within brackets.

_w_GRS_BMI_ = Weighted genetic risk score based on 97 BMI-associated SNPs.

_uw_GRS_BMI_ = Un-weighted genetic risk score based on 97 BMI-associated SNPs.

_w_GRS_Testosterone_ = Weighted genetic risk score based on 3 SNPs associated with serum testosterone.

_uw_GRS_T_ = Un-weighted genetic risk score based on 3 SNPs associated with serum testosterone.

*) Based on 96 instead of 97 SNPs.

Subjects known to use medications affecting sex hormones (testosterone, 5-alpha reductase inhibitors and antiandrogens) and/or who were surgically or chemically castrated, were excluded when this information was available. A more detailed description of the cohorts is available in the supplemental methods. All participants provided written, informed consent, and ethical permission was granted by the local research ethics committees for all participating studies (GOOD: local ethics committee at University of Gothenburg; MrOS Sweden: local ethics committees at Gothenburg University and Lund University; SHIP and SHIP Trend: local ethics committee of the University of Greifswald; Inter99: local ethics committee of Copenhagen County).

### Serum testosterone

GOOD and MrOS Sweden used a validated gas chromatography/mass spectroscopy (intra-assay CV, 2.9%; inter-assay CV, 3.4%) to measure serum T [20]. SHIP and Inter99 used immunoassays (intra-assay CV 8.9–13.3%, inter-assay CV 2.3%) [21] to measure serum T. A liquid chromatography-tandem mass spectrometry was used to measure serum T (intra-assay and inter-assay CVs<10%) in SHIP Trend [22].

### Serum SHBG

GOOD and MrOS Sweden used an immunoradiometric assay to measure serum SHBG (sex hormone-binding globuline; GOOD: intra-assay CV 3%, inter-assay CV 7%; MrOS Sweden: intra-assay CV <5.5%, inter-assay CV < 6.9%) [23]. SHIP used a competitive chemiluminescent enzyme immunoassays (inter-assay CV 6.6–7.7%) to measure serum SHBG [24]. Inter99 used a time-resolved immunofluorometric assay to measure serum SHBG (inter- and intra-assay CVs < 8%)[25].

### Genotyping and genetic risk scores

All cohorts except Inter99 used imputed genotype dosage SNP data (HapMap CEU r22) for the analyses. Inter99 had directly genotyped data (BeadChip) available for the BMI-associated SNPs and used KASPar from KBioscience for the three testosterone-associated SNPs. For details, please see supplemental methods.

The 97 BMI-associated SNPs identified in a recent large-scale GWAS on BMI were used to construct a weighted genetic risk score, _w_GRS_BMI,_ where weights were based on each SNP´s effect size with BMI in the meta-analysis by Locke et al [17]. For the primary analyses, the _w_GRS_BMI_ was used.

For T, a weighted T-decreasing genetic risk score (_w_GRS_T_) was developed based on three SNPs (rs6258, rs12150660, rs5934505) with weights identified in a recent large-scale GWAS on T [18].

Due to a partial overlap between the cohorts used by the original GWA studies identifying the SNPs and the present study an unweighted BMI-increasing genetic risk score (_uw_GRS_BMI_) and an unweighted T-decreasing genetic risk score (_uw_GRS_T_) were also developed. Please see supplemental methods for further details.

### Statistical analysis

The BMI data distribution was positively skewed and was therefore natural log transformed (Shapiro-Wilk test, p-value < 2.2e^-16^). Standardized residuals of ln BMI, serum SHBG and serum T (Z-scores) were calculated. An additive genetic model was used. Furthermore, although covariates are expected to be randomly distributed with respect to genotype, the associations between genotype and known confounders (age and smoking) were examined as this is a key assumption in MR analyses. In addition, due to the close connection between T and SHBG, the association between genotypes and SHBG were analyzed. Models with BMI as an outcome were adjusted for age, smoking and site, whereas models with serum T as an outcome were additionally adjusted for time of sampling. When evaluating a possible interaction effect between covariates and the genetic risk scores, an interaction term was added to the regression models.

The strength of the genetic risk scores as instruments was determined using the F statistic which was calculated as [F-stat = (n-2)*R^2^/(1-R^2^)], where R^2^ refers to the proportion of variance explained [26]. Two independent instrumental variables were used for each phenotype to assess pleiotropy. Linearity was assessed by adding a quadratic term to the regression analyses. The IV ratio method, using a two-stage least squares regression, was used to estimate the unconfounded causal effect of BMI on serum T and vice versa [27]. In addition, we also determined the causal effect of BMI on serum T after adjustment for serum SHBG as well as the causal effect of BMI on serum SHBG after adjustment for serum T.

In order to confirm the validity of our data, we also performed the combined analyses by pooling the samples. The effect on T as a result of a decline in BMI from 30 kg/m^2^ to 25 kg/m^2^ was estimated using pooled data and the obtained causal effect (the IV ratio) adjusted for age, smoking, cohort, site and blood sampling time. Since the IV ratio was calculated as standard deviations of T (SD(T)) per standard deviation of natural log transformed BMI (SD(log BMI)), the IV ratio was multiplied with SD(T) divided by SD(log BMI) to obtain the IV ratio in terms of T units per unit of log BMI. This was then multiplied with the difference between log 25 and log 30 to arrive at the difference in T levels given the decline in BMI from 30 kg/m^2^ to 25 kg/m^2^. In order to obtain the percentage change in T levels, the difference in T levels was then divided by the predicted T levels given a BMI of 30 kg/m^2^. In order to confirm our lack of evidence of a causal effect of T on BMI, we used summary statistics from the GIANT consortium (please see supplemental methods for details) [17, 28].

Power calculations for IV regression were based on a previously published analytical approach, using sample size, the observed association between phenotypes and the association between each phenotype and their genetic proxies [29]. To evaluate the ability to detect weaker effects on BMI using the _w_GRS_T_ and on serum T using _w_GRS_BMI_, power was also calculated based on half the observed association effect between BMI and serum T (1/2β).

Please see supplemental methods for further details on the statistical analysis.

## Results

### Phenotypic association between BMI and serum T: Observational estimates

**Fig 1:**
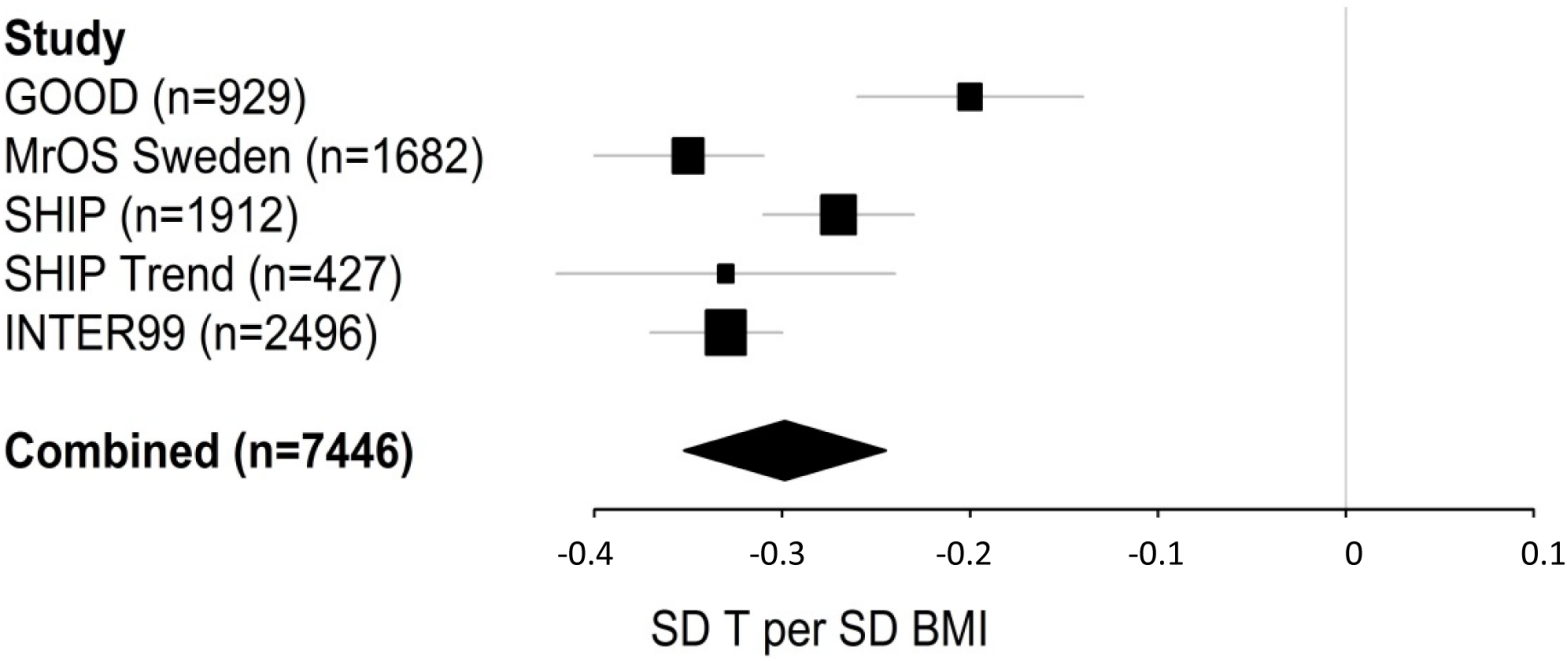
Observational estimates. An increased BMI is associated with a decreased serum testosterone (T). Linear regression models were used adjusting for age, smoking, site and time of day for blood sample, when applicable. The combined effect was calculated with random effect meta-analysis (I-squared = 82%, p = 0.0005) using all cohorts (n = 7446). Effect sizes are given in standard deviations (SD) of T per SD ln-transformed BMI. Horizontal bars represent 95% confidence intervals.

The observational association between BMI and serum T was significant in all cohorts after adjustment for age, smoking, site and blood sampling time, but slightly more pronounced in the MrOS cohort including the oldest men (mean age 75.4 years; -0.35 SD T per SD ln BMI) compared to the GOOD cohort including the youngest men (mean age 18.9 years; -0.20 SD T per SD ln BMI; Fig 1). In the meta-analysis of the five cohorts, we found evidence of heterogeneity (I-squared = 82%, p = 0.0005) among cohorts and therefore performed a random effect inverse-variance meta-analysis, demonstrating that a 1 SD increase in ln-transformed BMI was associated with a 0.30 SD decrease in serum T (95% CI -0.35–-0.24; Fig 1). A similar observational estimate was found in a pooled analysis of the five participating cohorts, revealing that 1 SD increase in ln-transformed BMI was associated with 0.30 SD decrease in serum T (95% CI: -0.33–-0.28).

### Validation of the instruments

Although both BMI and serum T were associated with age and smoking (p<0.05), the different GRSs evaluated in the present study (_w_GRS_BMI_, _uw_GRS_BMI_, _w_GRS_T_ and _uw_GRS_T_) were not associated with these two potential confounders (p>0.05; S1 Fig). This illustrates that these GRSs can be used as largely un-confounded instruments to assess the causality of the inverse association between BMI and serum T.

#### BMI SNPs

**Fig 2:**
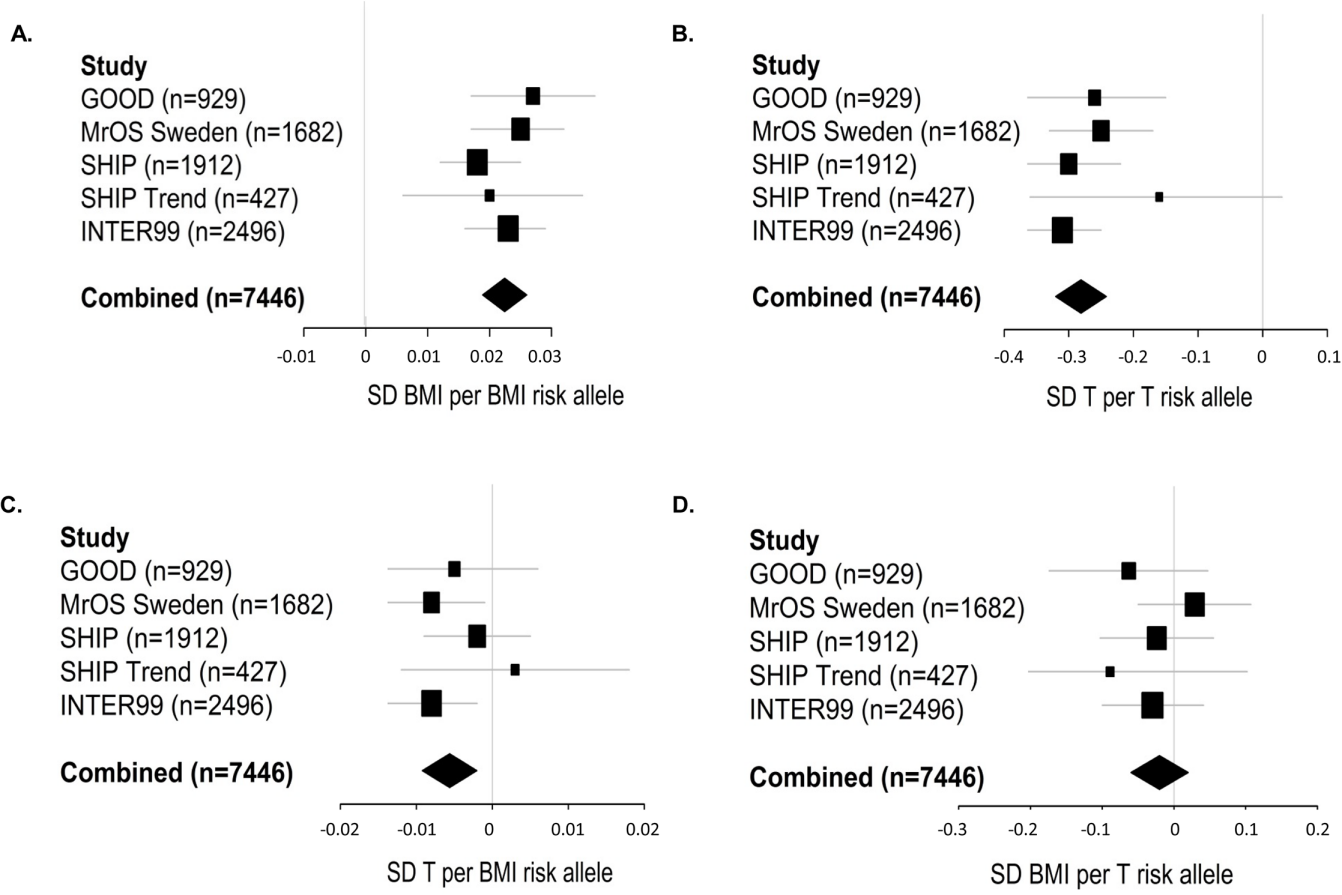
The genetic risk scores’ association with BMI and serum T. A) High weighted genetic risk score on BMI (_w_GRS_BMI_) is associated with high BMI. B) The weighted genetic risk score on serum testosterone (T; _w_GRS_T_) is inversely associated with serum T. C) The weighted genetic risk score on BMI (_w_GRS_BMI_) is inversely associated with serum testosterone (T). D) The weighted genetic risk score on serum T (_w_GRS_T_) is not associated with BMI. Linear regression models were used adjusting for age, smoking, site and time of day for blood sample, when applicable. The combined effect was calculated with fixed-effect meta-analysis (A: I-squared = 0%, p = 0.63; B: I-squared = 0%, p = 0.49; C: I-squared = 0%, p = 0.51; D: I-squared = 0%, p = 0.62) using all cohorts (n = 7446). Effect sizes are given in standard deviations (SD) of ln-transformed BMI per weighted BMI risk allele in A, SD of T per weighted T risk allele in B, SD of T per weighted BMI risk allele in C and SD of ln-transformed BMI per weighted T risk allele in D. Horizontal bars represent 95% confidence intervals.

Both _w_GRS_BMI_ and _uw_GRS_BMI_ were highly significantly associated with ln-transformed BMI in all cohorts individually and when combined in a meta-analytic approach (_w_*GRS*_*BMI*_ p = 4.2*10^−35^, R^2^ = 1.9%; _*uw*_*GRS*_*BMI*_ p = 5.0*10^−27^, R^2^ = 1.5%; Fig 2A, S1 Table). There was no evidence of heterogeneity in these analyses. The F-statistics, reflecting the strength of the instruments, were 147 and 111 for _w_GRS_BMI_ and _uw_GRS_BMI_, respectively (S1 Table). Similar significant associations were observed using pooling instead of a meta-analytic approach (S1 Table). The _w_GRS_BMI_ and _uw_GRS_BMI_ associations with BMI did not vary by age, smoking or serum T (p > 0.05 for all interaction terms in pooled analyses). No evidence of non-linearity was found for _w_GRS_BMI_ or _uw_GRS_BMI_. When the 97 SNPs included in the _w_GRS_BMI_ were evaluated separately, 16 SNPs were nominally (p<0.05) significantly associated with BMI. Three of these SNPs were still, after a conservative Bonferroni adjustment (p<0.05/97), significantly associated with BMI (S2 Table).

#### Testosterone SNPs

Both _w_GRS_T_ and _uw_GRS_T_ were highly significantly associated with serum T in the meta-analyzed combined data set (_*w*_*GRS*_*T*_ p = 4.7*10^−45^, R^2^ = 2.5%; _*uw*_*GRS*_*T*_ p = 3.9*10^−38^, R^2^ = 2.1%; Fig 2B, S3 Table). There was no evidence of heterogeneity in these analyses (Fig 2B). The F-statistics were 188 and 156 for _*w*_GRS_T_ and _uw_GRS_T_, respectively (S3 Table). Similar significant associations were observed using pooling instead of a meta-analytic approach (S3 Table). The _w_GRS_T_ and _uw_GRS_T_ associations with T did not vary by age, smoking or BMI (p values > 0.05 for all interaction terms in pooled analyses). No evidence of non-linearity was found for _w_GRS_T_ or _uw_GRS_T_. All three SNPs included in the _w_GRS_T_ were individually associated with serum T (p<0.05/3; S4 Table).

### Power analysis

The present study was adequately powered to detect a causal association between BMI and T if the strength of this association was equal to the observed association between serum BMI and T. In theory, we had approximately similar power to detect an association between BMI and T using the _w_GRS_BMI_ (95%) compared with an equal sized effect in the other direction using the _w_GRS_T_ (98%, n = 7446 subjects; S2 Fig).

### Evaluation of causal association using MR approach

#### BMI has a causal effect on serum testosterone

Both _*w*_GRS_BMI_ (p = 2.0*10^−3^) and _uw_GRS_BMI_ (p = 1.7*10^−3^) were significantly and inversely associated with serum T in the meta-analyzed combined cohort (Fig 2C; S1 Table). Similar significant associations were observed using pooling instead of a meta-analytic approach (S1 Table). After adjustment for BMI, none of these associations were still significant. The _*w*_GRS_BMI_ and _uw_GRS_BMI_ associations with T did not vary by age or smoking (p > 0.05 for interaction terms in pooled analyses). Interestingly, the two BMI SNPs that were most robustly associated with BMI were also significantly associated with serum T, but in the opposite direction (rs1558902 in the *FTO locus*, p = 4.0*10^−2^ and rs6567160 in the *M4CR locus*, p = 3.0*10^−3^; S2 Table). However, none of the SNPs were still significant after adjustment for BMI. IV analyses used to establish the direction and causality of the BMI-T association, revealed that 1 SD increase in ln-transformed BMI lead to a 0.25 (using _w_GRS_BMI_, p = 2.8*10^−3^) to 0.29 (using _uw_GRS_BMI_, p = 2.5*10^−3^) SD decrease in serum T (Table 2, S1 Table). There was no significant difference between β-coefficients from observational and IV analysis (_w_GRS_BMI_: p value 0.44, _uw_GRS_BMI_: p value 0.82).

**Table 2:** Summary of the coefficients used for IV ratio analyses.

IV	Risk Score with the Intermediate Trait	Risk score with the Outcome	IV Ratio	
	Coefficient (95% CI)	Coefficient (95% CI)	Coefficient (95% CI)	P value
_**w**_**GRS**_**BMI**_	0.022 (0.019–0.026)	-0.006 (-0.009–-0.002)	-0.25 (-0.42–-0.09)	0.003
_**uw**_**GRS**_**BMI**_	0.020 (0.016–0.023)	-0.006 (-0.009–-0.002)	-0.29 (-0.48–-0.10)	0.003
_**w**_**GRS**_**T**_	-0.281 (-0.320–-0.242)	-0.020 (-0.060–0.020)	0.07 (-0.56–0.70)	0.214
_**uw**_**GRS**_**T**_	-0.191 (-0.220–-0.162)	-0.004 (-0.034–0.025)	0.02 (-0.13–0.18)	0.777

IV ratio was calculated as the ratio between the genetic risk score's association with the outcome and intermediate trait (n = 7446).

Coefficients for intermediate trait and outcome are given as SD per unit in genetic risk score.

_w_GRS_BMI_ = Weighted genetic risk score on BMI.

_uw_GRS_BMI_ = Un-weighted genetic risk score on BMI

_w_GRS_T_ = Weighted genetic risk score on serum testosterone.

_uw_GRST_T_ = Un-weighted genetic risk score on serum testosterone.

IV ratio is expressed in SD testosterone per SD ln-transformed BMI for _w_GRS_BMI_ and _uw_GRS_BMI_, while it is expressed in SD ln-transformed BMI per SD testosterone for _w_GRS_T_ and _uw_GRS_T_.

**Fig 3:**
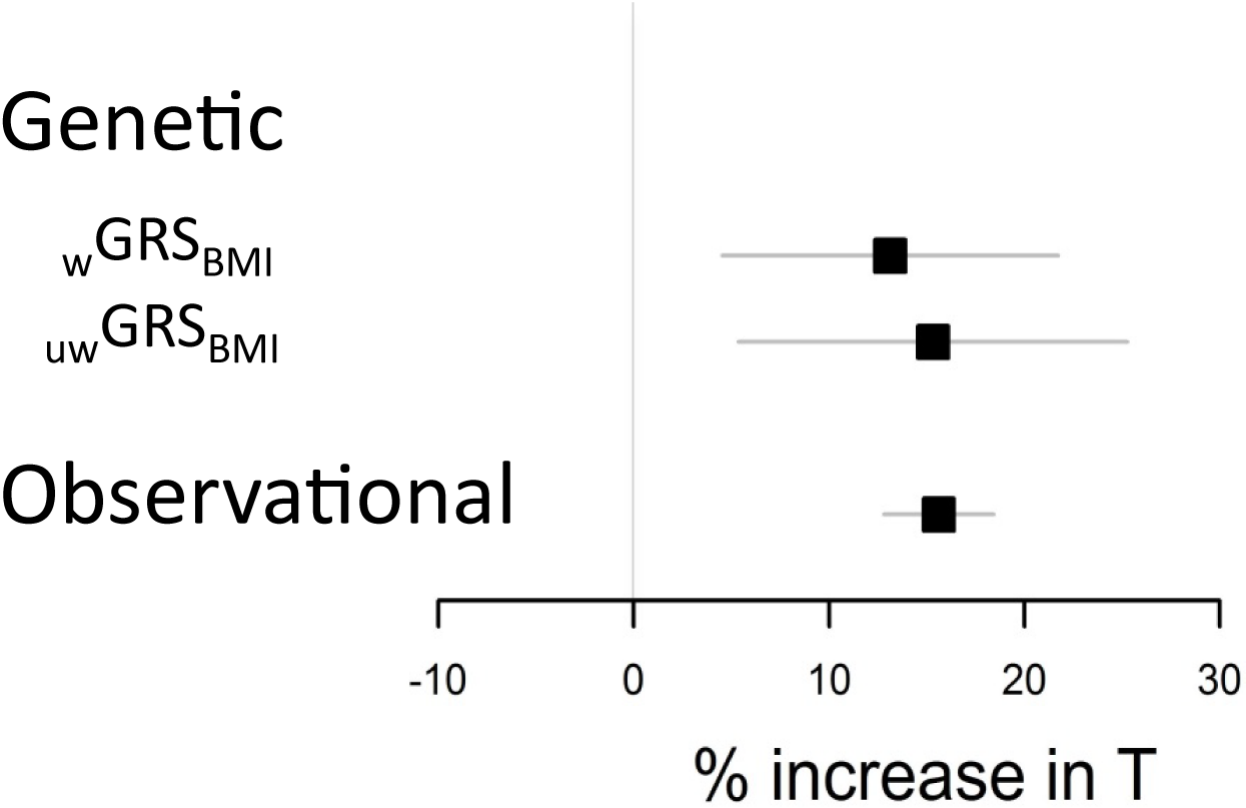
Genetic and observational estimates of the influence of altering BMI on serum testosterone (T). Effect sizes are given in percent change in serum T based on a decrease in BMI from 30 (cut off for obesity) to 25 (cut off for overweight) kg/m^2^ (n = 7446). _w_GRS_BMI_ = weighted genetic risk score on BMI. _uw_GRS_BMI_ = un-weighted genetic risk score on BMI. Horizontal bars represent 95% confidence intervals.

Notably, for a body weight reduction altering the BMI from 30 (cut off for obesity) to 25 (cut off for overweight) kg/m^2^, the effect would equal a 13% (using IV ratio from _w_GRS_BMI_) to 15% (using IV ratio from _uw_GRS_BMI_) increase in serum T while a corresponding observational effect would equal a 16% increase in serum T (Fig 3). To reduce the risk of pleiotropy, we next evaluated two separate independent genetic instruments (rs1558902 from the *FTO locus* and a weighted risk score based on the remaining 96 SNPs). These two independent genetic instruments had similar point estimates of the causal effect of BMI on serum T (S5 Table).

#### No evidence of a causal effect of serum testosterone on BMI

Neither the _*w*_GRS_T_ nor the _uw_GRS_T_ was associated with BMI (Fig 2D, S3 Table). Furthermore, the IV ratio analyses provided no evidence for a causal effect of serum T on BMI (Table 2, S3 Table). For the two autosomal SNPs it was possible to use the GIANT consortium, including up to 104349 men, to evaluate the causal effect of serum T on BMI in a larger setting. However, no significant evidence was found supporting that any of these T SNPs were associated with BMI individually, or when combined as a genetic risk score (S6 Table).

Due to the complexity of the T synthesis and bio-availability we also performed sub-analyses using two separate GRSs based on SNPs located within, or outside, the *SHBG locus*. Although both these two separate T GRSs were robustly associated with serum T, no evidence of significant associations between these two separate T GRSs and BMI was observed (S7 Table).

#### Genetic risk scores for BMI and serum T in relation to serum SHBG

Since serum SHBG is a known major determinant of serum total T, we also tested the association between serum SHBG and the weighted genetic risk scores for BMI (_w_GRS_BMI_) and serum T (_w_GRS_T_). _w_GRS_BMI_ was inversely associated with serum SHBG in the pooled combined cohort (S3 Fig). However, after adjustment for serum T _w_GRS_BMI_ was not significantly associated with SHBG (combined cohort: p = 0.59). _w_GRS_T_ was robustly associated with serum SHBG (combined cohort: p = 2.7*10^−49^, S3 Fig). Interestingly, the association between _w_GRS_BMI_ and serum T remained significant (p = 0.02) after adjustment for serum SHBG in the pooled combined cohort.

## Discussion

Obesity is associated with low serum T in men, but the direction of the association and the potentially causal effect is still debated. Herein, we presented evidence that higher BMI leads to lower serum T. Conversely, our analyses provided no evidence for a causal role of serum T on BMI. These results suggest that although increases in T status are not likely to help with weight regulation in the general male population, low serum T could contribute to the adverse health effects associated with obesity in men. Our study highlights the importance of considering obesity as a risk factor for low serum T in men with implications on the possible targeting of relevant health promotion strategies.

We evaluated the relationship between BMI and serum T using a bi-directional MR analysis which is free of reverse causation. Our genetic instruments in these analyses were derived from the most recent large-scale GWAS-meta-analyses on serum T and BMI [17, 18]. Three key assumptions underlie the MR randomization approach: the genotypes are randomized; the genetic variants considered as instruments affect the outcome only by modifying the biomarker—that is, these variants have no pleiotropic effects on the outcome; and the genotype is independent of confounders [27, 30, 31]. We did not detect any violations of the assumptions underlying MR as far as they could be tested. For instance, although both BMI and T were associated with age and smoking, the different GRSs used in the present study were not associated with these two potential confounders. It could be argued that as the biological functions for some of the used BMI-associated SNPs are yet to be established [17, 28], there could be alternative biological pathways explaining their association with BMI. However, using as many as 97 recently reported independent SNPs to index BMI, we were able to minimize the risk of shared pleiotropy and linkage-disequilibrium-induced confounding pleiotropic effects [32, 33]. Moreover, the use of two separate independent genetic instruments with similar point estimates of the causal effect of BMI on serum T further reduced the risk of pleiotropy [32, 33].

In the present study, there was a small overlap (less than 2%), between the cohorts used in the present study and the study by Locke et al that identified the BMI-associated SNPs[17]. As a consequence, there is a chance that the causal estimate calculated using the _w_GRS_BMI_ could be biased. It is, however, highly unlikely that this has had a major impact on the results for two reasons. Firstly, very similar results were obtained using an unweighted genetic risk score that avoids the potential bias from the use of internal weights. Secondly, similar results were also obtained using a genetic risk score based solely on the SNP in the FTO gene, which is a gene that has been found to be associated with BMI in numerous studies, where none of the cohorts used in the present study were included [34–36]. The validity of our findings was strengthened by the fact that very similar results were obtained for both weighted and un-weighted genetic risk scores and when combined estimates were calculated using pooling and a meta-analytic approach. Also, as we included participants at random and consecutively from the general population, the potential for selection bias is minimal.

Cross-sectional data from the large European Male Aging Study (EMAS) revealed that BMI was the strongest determinant of serum T and obese men (BMI > 30 kg/m^2^) had as much as 1.47 ng/ml lower serum T compared with normal weight subjects (BMI < 25 kg/m^2^; [1]). The main finding in the present study is our results supporting a causal effect of BMI on serum T. The magnitude of this effect is similar to the observational association. Each SD genetically instrumented increase in BMI was associated with a 0.25 SD decrease in serum T. For a body weight reduction altering the BMI from 30 to 25 kg/m^2^, the effect would equal a 13–15% increase in serum T. This evidence of a causative effect of BMI on serum T is supported by a recent meta-analysis of the impact of body weight reduction on serum T, revealing that body weight reductions as a result of both low-calorie diet and bariatric surgery are associated with significantly increased serum T [37]. Notably, this meta-analysis demonstrated that the degree of body weight loss is a robust determinant of the increase in serum T. The mean percent body weight loss at endpoint was 10% in low-calorie diet studies and 32% in bariatric surgery studies and the corresponding increases in serum T levels were 0.83 ng/ml and 2.54 ng/ml, respectively. It was even proposed that normalization of T levels is a possible mechanism contributing to the beneficial health effect of bariatric surgery in morbid obesity [37].

Although the present study provides compelling evidence that obesity reduces serum T levels, it cannot determine the mechanism for this causal effect which may occur at several levels of the hypothalamus-pituitary-gonadal axis. Obese subjects often have reduced gonadotropin concentrations indicating that the primary effect is mediated at the hypothalamus-pituitary level rather than at the testicular level [38]. Not only serum T but also the gonadotropins FSH and LH were increased by body weight reduction, suggesting that body weight loss reverses obesity-associated hypogonadotropic hypogonadism [37]. It is well known that in morbidly obese men, LH levels and pulse amplitude are attenuated when compared with normal-weight controls [39, 40]. Thus high BMI seems to repress serum T mainly via central inhibitory effects on gonadotropin secretion [38, 41]. The exact mechanism for this inhibitory effect of high BMI/obesity on gonadotropin secretion is unclear but might include insulin resistance, inflammatory mediators, leptin, hypothalamic kisspeptin affecting gonadotropin -releasing hormone (GnRH) secretion and/or increased aromatase activity resulting in elevated estradiol levels which in turn augment negative feed-back regulation [7, 37, 38, 42–44].

Notably, the two SNPs most robustly associated with BMI in the present study are also significantly associated with serum T but in the opposite direction. The underlying loci of these two SNPs, *FTO* and *MC4R*, are well established obesity-related loci [35, 36, 45]. The *FTO* locus has previously been reported to be associated with affected androgen levels, polycystic ovary syndrome susceptibility and age at menarche in females while its impact on serum T and T-related diseases in men is unknown and warrants further investigations [46, 47].

Our study highlights the importance of considering obesity as a risk factor for low testosterone concentrations in men with implications for its possible targeting in relevant health promotion strategies. Our finding supports the notions that male hypogonadism can be considered as one of the many adverse consequences of overweight and obesity and that body weight loss and lifestyle interventions should be the first approach offered to obese men with low serum T [48]. Based on the described significant secular trends of reduced serum T and increased BMI in men [25, 49] and the present evidence of a causative effect of BMI on T, we propose that successful population level interventions reverting the obesity epidemic might also lead to a reversal of the secular trend of reduction in serum T.

Our analyses provided no evidence for a causal role of serum T on BMI. It could be argued that if a possible effect of serum T on BMI would be substantially less pronounced than the observational association between T and BMI, then our study would not be adequately powered. However, the use of the GIANT consortium to test the association between T-related autosomal SNPs and BMI in a much larger sample including 104349 men also failed to identify a causal role of T on BMI. While the MR approach enables the approximation of life-long differences in average concentrations, genetic markers do not examine the influences arising from the extremes of non-linear distributions [28, 50]. Consequently, we cannot discount a possible causative effect of severe T-deficiency on BMI. Due to the complexity of the T synthesis and bio-availability we performed sub-analyses using two separate GRSs based on SNPs located within, or outside, the *SHBG locus*. SHBG is the dominant high-affinity binding protein for serum T and its primary function is to bind and transport steroids in the blood to access target tissues and to determine the bioavailable fraction. Although both these two separate T GRSs were robustly associated with serum T, no evidence of significant associations between these two separate T GRSs and BMI was observed.

Serum SHBG is a major well-known determinant of total serum T. In fact, in the cohorts included in this study SHBG explained about 26% of the total variance in serum T. This close connection between serum T and SHBG makes it difficult to discriminate between an effect directly on serum T and an effect mediated via SHBG. In the present study, we found evidence of a causal effect of BMI on serum T, but the BMI-based genetic risk score was also associated with serum SHBG. When serum T was added as a covariate to the analysis, the association between the BMI-based genetic risk scores and SHBG was no longer significant. However, in the opposite analysis where serum T was the outcome and SHBG the covariate, _w_GRS_BMI_ was significantly associated with serum T, supporting a SHBG-independent effect of BMI on serum T. Two of the three SNPs constituting the genetic risk score for BMI reside in the SHBG locus and have previously been reported to associate with SHBG (18). In line with this, both _w_GRS_T_ and _uw_GRS_T_ were significantly associated with SHBG in the present study.

The participant data used in the present study were unrelated men with European ancestry, limiting the generalizability of the observed findings to other ethnic groups. However, this also means that a major influence from population stratification is unlikely.

In conclusion, our results suggest that there is a causal effect of BMI on serum T in men. Population level interventions to reduce BMI are expected to increase serum T in men.

## Supporting information

S1 Fig The genetic risk scores are not associated with age or smoking. (TIF)

S2 Fig Power analysis. (TIF)

S3 Fig Associations between genetic risk scores and SHBG. (TIF)

S1 Table Summary of the IV analyses using the weighted and un-weighted genetic risk scores for BMI. (DOCX)

S2 Table Summary of the association for the 97 BMI-associated SNPs with BMI and testosterone. (DOCX)

S3 Table Summary of the IV analyses using the weighted and un-weighted genetic risk scores for testosterone. (DOCX)

S4 Table Summary of the association for the three testosterone-associated SNPs with testosterone and BMI. (DOCX)

S5 Table Summary of the IV analyses using independent genetic risk scores for BMI. (DOCX)

S6 Table Associations between testosterone SNPs and BMI when evaluated in the Giant consortium. (DOCX)

S7 Table Summary of the IV analyses using genetic risk scores for testosterone. (DOCX)

S1 File Supplemental methods. (DOCX)
