# Supplementary figures and images for: Causal relationship between obesity and serum testosterone status in men: A bi-directional mendelian randomization analysis
Source: PLoS One. 2017 Apr 27;12(4):e0176277. doi: 10.1371/journal.pone.0176277 (PMC5407807; doi:10.1371/journal.pone.0176277)

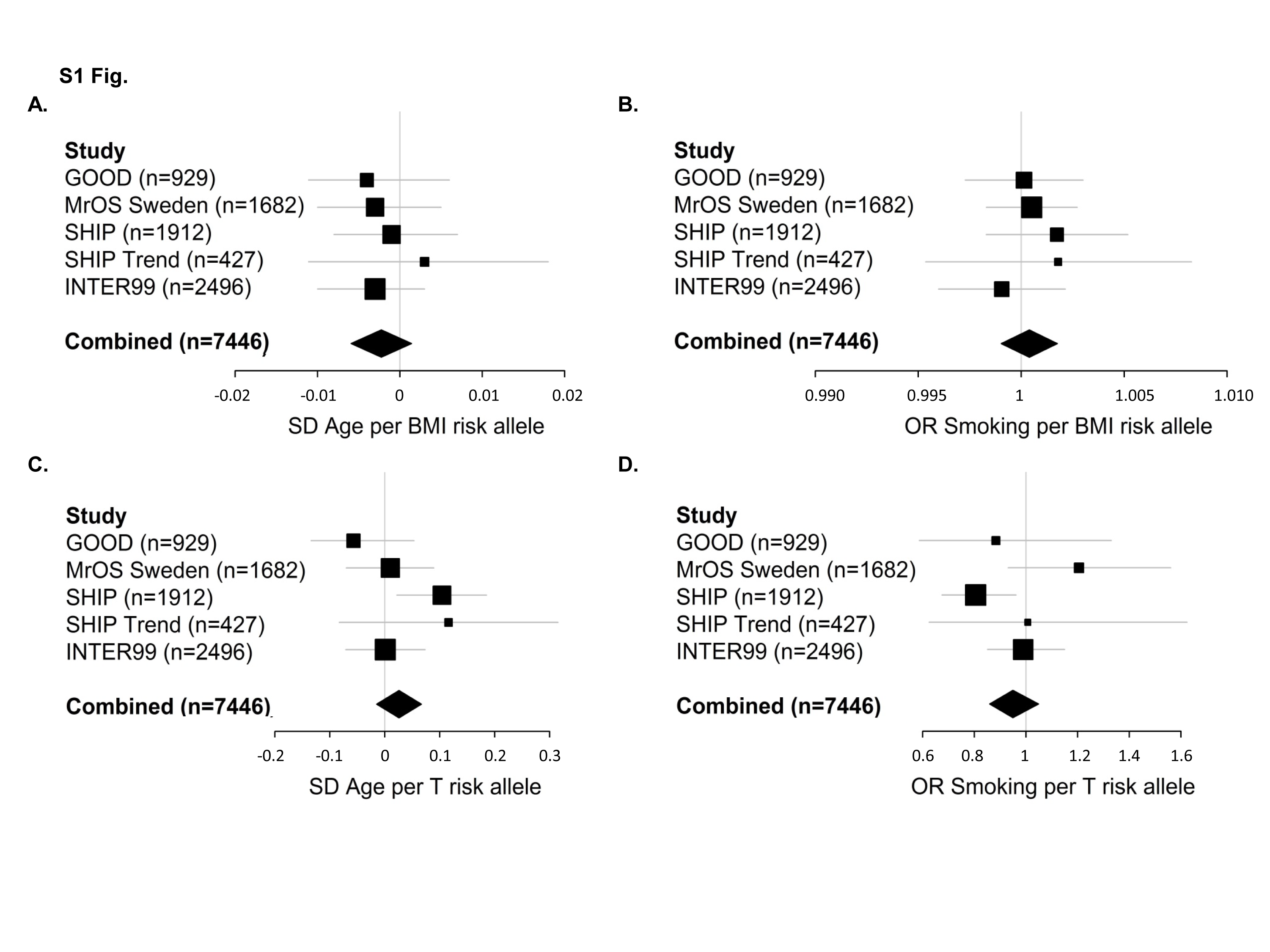

Supplement: S1 Fig — (TIF) [file pone.0176277.s001.tif]

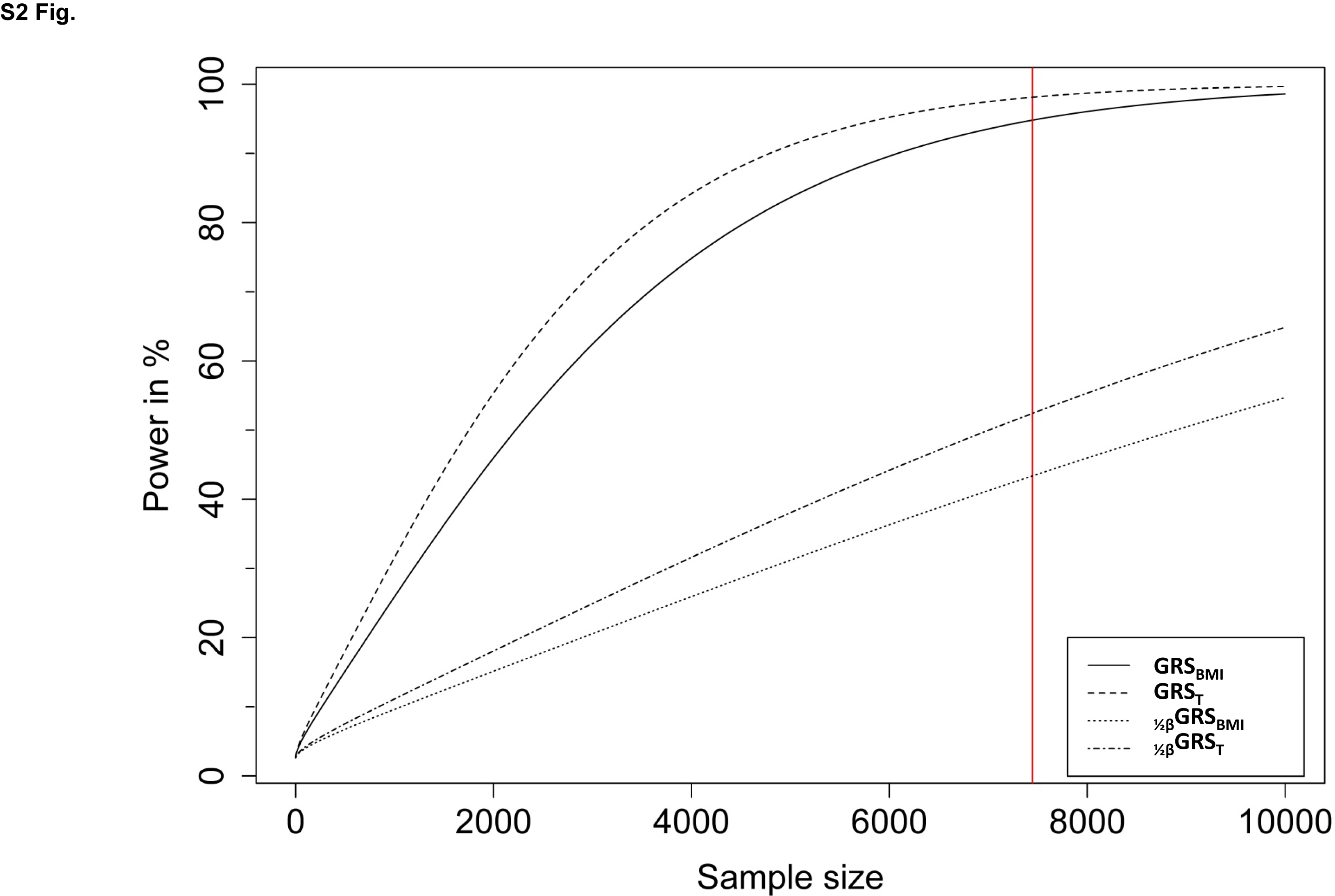

Supplement: S2 Fig — (TIF) [file pone.0176277.s002.tif]

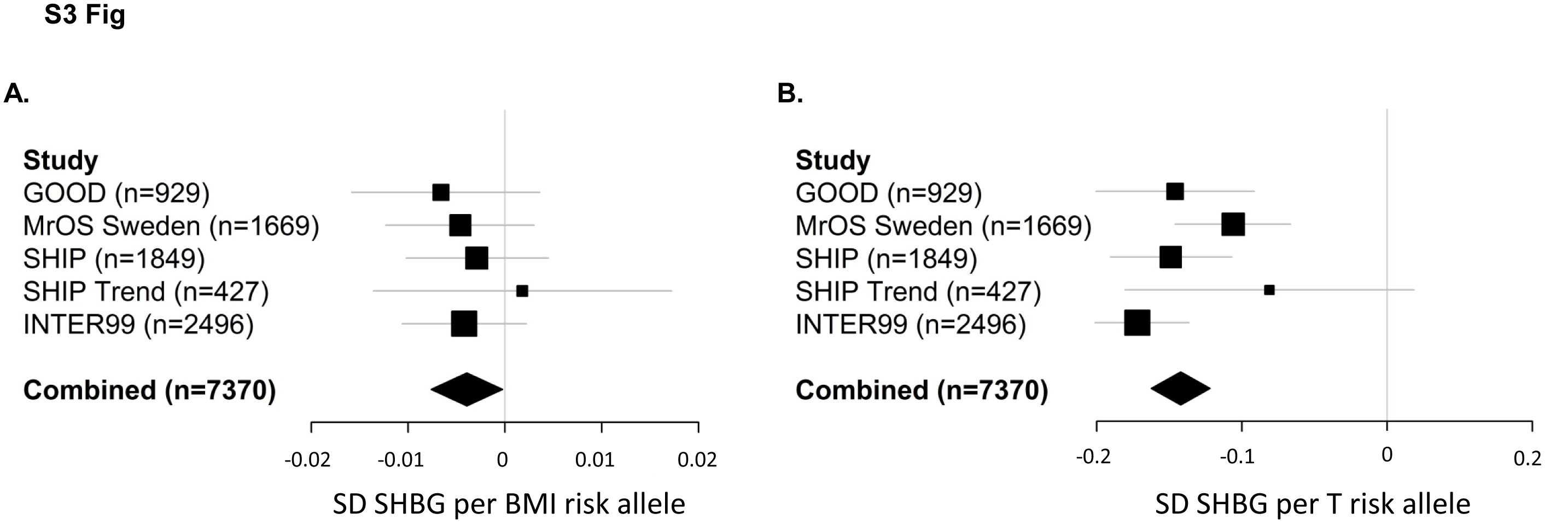

Supplement: S3 Fig — (TIF) [file pone.0176277.s003.tif]
