# Supplementary material for: Causal relationship between obesity and serum testosterone status in men: A bi-directional mendelian randomization analysis
Source: PLoS One. 2017 Apr 27;12(4):e0176277. doi: 10.1371/journal.pone.0176277 (PMC5407807; doi:10.1371/journal.pone.0176277)
Supplement: S1 Table — (DOCX) [file pone.0176277.s004.docx]

| **S1 Table. Summary of the IV analyses using the weighted and un-weighted genetic risk scores for BMI.** | | | | | | | | | |  |  |  |  |
| --- | --- | --- | --- | --- | --- | --- | --- | --- | --- | --- | --- | --- | --- |
|  |  | | | | |  |  |  |  |  |  |  |  |
|  |  |  | **1st Stage: GRS vs BMI** | | | | **2nd stage: GRS vs T** | | | | **IV** | | |
| Genetic risk score | Cohort | F | BETA | SE | p | R^2^ | BETA | SE | p | R^2^ | IV | SE | p |
| **_w_GRS_BMI_** | GOOD (n=929) | 28 | 0.027 | 0.005 | 1.6E-07 | 2.9% | -0.005 | 0.005 | 3.8E-01 | 0.1% | -0.17 | 0.19 | 3.8E-01 |
|  | MrOS Sweden (n=1682) | 42 | 0.025 | 0.004 | 7.1E-11 | 2.4% | -0.008 | 0.004 | 3.2E-02 | 0.3% | -0.33 | 0.16 | 4.1E-02 |
|  | SHIP (n=1912) | 25 | 0.018 | 0.004 | 2.1E-07 | 1.3% | -0.002 | 0.004 | 5.1E-01 | 0.0% | -0.13 | 0.20 | 5.2E-01 |
|  | SHIP Trend (n=427) | 6 | 0.020 | 0.007 | 8.2E-03 | 1.5% | 0.003 | 0.008 | 7.0E-01 | 0.0% | 0.15 | 0.39 | 7.0E-01 |
|  | INTER99 (n=2496) | 49 | 0.023 | 0.003 | 1.8E-12 | 1.9% | -0.008 | 0.003 | 8.8E-03 | 0.3% | -0.37 | 0.15 | 1.4E-02 |
|  |  |  |  |  |  |  |  |  |  |  |  |  |  |
|  | **Meta-analysed combined (n=7446)** | **147** | **0.022** | **0.002** | **4.2E-35** | **1.9%** | **-0.006** | **0.002** | **2.0E-03** | **0.1%** | **-0.25** | **0.08** | **2.8E-03** |
|  |  |  |  |  |  |  |  |  |  |  |  |  |  |
|  | **Pooled combined (n=7446)** | **120** | **0.020** | **0.002** | **1.3E-34** | **1.6%** | **-0.006** | **0.002** | **1.5E-03** | **0.1%** | **-0.28** | **0.09** | **2.1E-03** |
|  |  |  |  |  |  |  |  |  |  |  |  |  |  |
| **_uw_GRS_BMI_** |  |  |  |  |  |  |  |  |  |  |  |  |  |
|  | GOOD (n=929) | 21 | 0.023 | 0.005 | 5.5E-06 | 2.2% | -0.009 | 0.005 | 7.6E-02 | 0.3% | -0.39 | 0.24 | 9.8E-02 |
|  | MrOS Sweden (n=1682) | 34 | 0.022 | 0.004 | 3.5E-09 | 2.0% | -0.009 | 0.004 | 2.1E-02 | 0.3% | -0.39 | 0.18 | 3.2E-02 |
|  | SHIP (n=1912) | 21 | 0.017 | 0.004 | 2.7E-06 | 1.1% | -0.002 | 0.004 | 5.9E-01 | 0.0% | -0.11 | 0.22 | 6.0E-01 |
|  | SHIP Trend (n=427) | 5 | 0.017 | 0.007 | 2.5E-02 | 1.1% | 0.003 | 0.008 | 6.5E-01 | 0.0% | 0.21 | 0.46 | 6.5E-01 |
|  | INTER99 (n=2496) | 33 | 0.019 | 0.003 | 5.1E-09 | 1.3% | -0.007 | 0.003 | 2.9E-02 | 0.2% | -0.37 | 0.18 | 4.1E-02 |
|  |  |  |  |  |  |  |  |  |  |  |  |  |  |
|  | **Meta-analysed combined (n=7446)** | **111** | **0.020** | **0.002** | **5.0E-27** | **1.5%** | **-0.006** | **0.002** | **1.7E-03** | **0.1%** | **-0.29** | **0.10** | **2.5E-03** |
|  |  |  |  |  |  |  |  |  |  |  |  |  |  |
|  | **Pooled combined (n=7446)** | **91** | **0.018** | **0.002** | **1.1E-26** | **1.2%** | **-0.006** | **0.002** | **1.4E-03** | **0.1%** | **-0.33** | **0.11** | **2.1E-03** |
| IV is the instrumental variable ratio which was calculated as the ratio between the association of the weighted (_w_GRS_BMI_) and un-weighted BMI genetic risk score (_uw_GRS_BMI_) with z-scored serum testosterone and the association between the genetic risk scores and z-scored ln-transformed BMI. Linear regression models were adjusted for age, smoking, site and time of day for blood samples, when applicable. Beta and se are expressed in standard deviations per unit of GRS. F is the F statistics. R^2^ is the variance explained. T = serum testosterone. Both fixed effect meta-analysis (Meta-analysed combined) and analyses with pooled data (Pooled combined) were used, yielding similar results. | | | | | | | | | | | | | |
