# Supplementary material for: Causal relationship between obesity and serum testosterone status in men: A bi-directional mendelian randomization analysis
Source: PLoS One. 2017 Apr 27;12(4):e0176277. doi: 10.1371/journal.pone.0176277 (PMC5407807; doi:10.1371/journal.pone.0176277)
Supplement: S2 Table — (DOCX) [file pone.0176277.s005.docx]

| **S2 Table. Summary of the association for the 97 BMI-associated SNPs with BMI and testosterone.** | | | | | | | | | |  |  |  |  |
| --- | --- | --- | --- | --- | --- | --- | --- | --- | --- | --- | --- | --- | --- |
|  |  |  |  |  |  |  |  |  |  |  |  |  |  |
|  |  | Position | Nearest | Effect | Other | Effect Allele | **BMI** | | | **Testosteone** | | |  |
| SNP | Chr | (bp) | Gene | Allele | Allele | Frequency | Beta_BMI_ | SE_BMI_ | P value_BMI_ | Beta_T_ | SE_T_ | P value_T_ | N |
|  |  |  |  |  |  |  |  |  |  |  |  |  |  |
| rs1558902 | 16 | 52361075 | FTO | A | T | 0.41 | 0.084 | 0.016 | **2.6E-07** | -0.033 | 0.016 | 4.0E-02 | 7446 |
| rs6567160 | 18 | 55980115 | MC4R | C | T | 0.24 | 0.071 | 0.019 | **1.5E-04** | -0.056 | 0.019 | 3.0E-03 | 7446 |
| rs7138803 | 12 | 48533735 | BCDIN3D | A | G | 0.41 | 0.057 | 0.016 | **4.6E-04** | 0.009 | 0.016 | 5.8E-01 | 7446 |
| rs2365389 | 3 | 61211502 | FHIT | C | T | 0.57 | 0.049 | 0.016 | 2.3E-03 | -0.028 | 0.016 | 8.2E-02 | 7446 |
| rs2121279 | 2 | 142759755 | LRP1B | T | C | 0.14 | 0.067 | 0.023 | 3.6E-03 | -0.029 | 0.023 | 2.0E-01 | 7446 |
| rs17024393 | 1 | 109956211 | GNAT2 | C | T | 0.04 | 0.115 | 0.042 | 6.0E-03 | -0.051 | 0.042 | 2.2E-01 | 7446 |
| rs4787491 | 16 | 29922838 | INO80E | G | A | 0.51 | 0.044 | 0.016 | 6.5E-03 | -0.032 | 0.016 | 4.2E-02 | 7446 |
| rs16851483 | 3 | 142758126 | RASA2 | T | G | 0.06 | 0.088 | 0.034 | 9.6E-03 | 0.028 | 0.034 | 4.1E-01 | 7446 |
| rs11030104 | 11 | 27641093 | BDNF | A | G | 0.78 | 0.050 | 0.020 | 1.0E-02 | -0.002 | 0.020 | 9.2E-01 | 7446 |
| rs12940622 | 17 | 76230166 | RPTOR | G | A | 0.56 | 0.041 | 0.016 | 1.2E-02 | 0.003 | 0.016 | 8.5E-01 | 7446 |
| rs13021737 | 2 | 622348 | TMEM18 | G | A | 0.83 | 0.054 | 0.022 | 1.3E-02 | 0.022 | 0.022 | 3.2E-01 | 7446 |
| rs10132280 | 14 | 24998019 | STXBP6 | C | A | 0.68 | 0.040 | 0.018 | 2.4E-02 | -0.013 | 0.018 | 4.7E-01 | 7446 |
| rs29941 | 19 | 39001372 | KCTD15 | G | A | 0.68 | 0.038 | 0.017 | 2.9E-02 | -0.027 | 0.017 | 1.2E-01 | 7446 |
| rs17203016 | 2 | 207963763 | CREB1 | G | A | 0.21 | 0.041 | 0.020 | 4.3E-02 | 0.015 | 0.020 | 4.6E-01 | 7446 |
| rs13107325 | 4 | 103407732 | SLC39A8 | T | C | 0.06 | 0.074 | 0.037 | 4.3E-02 | 0.002 | 0.037 | 9.5E-01 | 7446 |
| rs2112347 | 5 | 75050998 | POC5 | T | G | 0.63 | 0.034 | 0.017 | 4.3E-02 | -0.011 | 0.017 | 5.2E-01 | 7446 |
| rs7715256 | 5 | 153518086 | GALNT10 | G | T | 0.42 | 0.032 | 0.016 | 5.1E-02 | 0.004 | 0.016 | 8.2E-01 | 7446 |
| rs17001654 | 4 | 77348592 | SCARB2 | G | C | 0.14 | 0.044 | 0.023 | 6.0E-02 | -0.009 | 0.023 | 7.1E-01 | 7446 |
| rs2033529 | 6 | 40456631 | TDRG1 | G | A | 0.29 | 0.033 | 0.018 | 6.6E-02 | -0.005 | 0.018 | 7.7E-01 | 7446 |
| rs11126666 | 2 | 26782315 | KCNK3 | A | G | 0.27 | 0.034 | 0.019 | 7.4E-02 | -0.003 | 0.019 | 8.9E-01 | 7446 |
| rs3101336 | 1 | 72523773 | NEGR1 | C | T | 0.60 | 0.029 | 0.016 | 7.5E-02 | -0.002 | 0.016 | 9.2E-01 | 7446 |
| rs16951275 | 15 | 65864222 | MAP2K5 | T | C | 0.78 | 0.034 | 0.019 | 7.7E-02 | 0.004 | 0.019 | 8.3E-01 | 7446 |
| rs2080454 | 16 | 47620091 | CBLN1 | C | A | 0.41 | 0.029 | 0.016 | 7.7E-02 | -0.024 | 0.016 | 1.5E-01 | 7446 |
| rs9641123 | 7 | 93035668 | CALCR | C | G | 0.43 | 0.034 | 0.021 | 9.5E-02 | 0.006 | 0.021 | 7.8E-01 | 4950 |
| rs7164727 | 15 | 70881044 | LOC100287559 | T | C | 0.69 | 0.029 | 0.017 | 9.7E-02 | -0.024 | 0.017 | 1.6E-01 | 7446 |
| rs10938397 | 4 | 44877284 | GNPDA2 | G | A | 0.42 | 0.027 | 0.016 | 9.8E-02 | -0.020 | 0.016 | 2.2E-01 | 7446 |
| rs205262 | 6 | 34671142 | C6orf106 | G | A | 0.28 | 0.030 | 0.018 | 1.0E-01 | 0.003 | 0.018 | 8.7E-01 | 7446 |
| rs3810291 | 19 | 52260843 | ZC3H4 | A | G | 0.66 | 0.028 | 0.018 | 1.2E-01 | -0.005 | 0.018 | 8.0E-01 | 7446 |
| rs2207139 | 6 | 50953449 | TFAP2B | G | A | 0.17 | 0.033 | 0.021 | 1.2E-01 | -0.032 | 0.021 | 1.3E-01 | 7446 |
| rs2820292 | 1 | 200050910 | NAV1 | C | A | 0.55 | 0.025 | 0.016 | 1.2E-01 | -0.004 | 0.016 | 8.1E-01 | 7446 |
| rs543874 | 1 | 176156103 | SEC16B | G | A | 0.20 | 0.030 | 0.020 | 1.4E-01 | 0.011 | 0.020 | 5.9E-01 | 7446 |
| rs13191362 | 6 | 162953340 | PARK2 | A | G | 0.88 | 0.037 | 0.025 | 1.4E-01 | 0.018 | 0.025 | 4.5E-01 | 7446 |
| rs4740619 | 9 | 15624326 | C9orf93 | T | C | 0.57 | 0.023 | 0.016 | 1.4E-01 | 0.004 | 0.016 | 8.2E-01 | 7446 |
| rs2245368 | 7 | 76446079 | PMS2L11 | C | T | 0.17 | 0.034 | 0.023 | 1.4E-01 | -0.026 | 0.023 | 2.7E-01 | 7446 |
| rs7599312 | 2 | 213121476 | ERBB4 | G | A | 0.70 | 0.026 | 0.018 | 1.5E-01 | 0.013 | 0.018 | 4.8E-01 | 7446 |
| rs492400 | 2 | 219057996 | USP37 | C | T | 0.42 | 0.023 | 0.016 | 1.5E-01 | 0.019 | 0.016 | 2.3E-01 | 7446 |
| rs12566985 | 1 | 74774781 | FPGT-TNNI3K | G | A | 0.43 | 0.023 | 0.016 | 1.5E-01 | -0.011 | 0.016 | 5.1E-01 | 7446 |
| rs9400239 | 6 | 109084356 | FOXO3 | C | T | 0.70 | 0.025 | 0.018 | 1.5E-01 | 0.008 | 0.017 | 6.4E-01 | 7446 |
| rs12401738 | 1 | 78219349 | FUBP1 | A | G | 0.36 | 0.023 | 0.017 | 1.7E-01 | 0.022 | 0.017 | 1.9E-01 | 7446 |
| rs6477694 | 9 | 110972163 | EPB41L4B | C | T | 0.40 | 0.022 | 0.016 | 1.8E-01 | -0.026 | 0.016 | 1.1E-01 | 7446 |
| rs11727676 | 4 | 145878514 | HHIP | T | C | 0.91 | 0.042 | 0.032 | 1.9E-01 | -0.023 | 0.031 | 4.7E-01 | 7446 |
| rs1167827 | 7 | 75001105 | HIP1 | G | A | 0.57 | 0.022 | 0.017 | 1.9E-01 | -0.001 | 0.017 | 9.6E-01 | 7446 |
| rs2287019 | 19 | 50894012 | QPCTL | C | T | 0.79 | 0.027 | 0.021 | 1.9E-01 | 0.001 | 0.021 | 9.5E-01 | 7446 |
| rs10182181 | 2 | 25003800 | ADCY3 | G | A | 0.47 | 0.021 | 0.016 | 2.0E-01 | -0.014 | 0.016 | 3.8E-01 | 7446 |
| rs758747 | 16 | 3567359 | NLRC3 | T | C | 0.26 | 0.024 | 0.019 | 2.1E-01 | -0.001 | 0.019 | 9.6E-01 | 7446 |
| rs7903146 | 10 | 114748339 | TCF7L2 | C | T | 0.73 | 0.023 | 0.018 | 2.1E-01 | 0.013 | 0.018 | 4.6E-01 | 7446 |
| rs3888190 | 16 | 28796987 | ATP2A1 | A | C | 0.42 | 0.020 | 0.016 | 2.1E-01 | -0.019 | 0.016 | 2.5E-01 | 7446 |
| rs11847697 | 14 | 29584863 | PRKD1 | T | C | 0.04 | 0.049 | 0.042 | 2.4E-01 | -0.058 | 0.042 | 1.6E-01 | 7446 |
| rs1808579 | 18 | 19358886 | C18orf8 | C | T | 0.52 | 0.018 | 0.016 | 2.6E-01 | -0.070 | 0.016 | **1.4E-05** | 7446 |
| rs13078960 | 3 | 85890280 | CADM2 | G | T | 0.19 | 0.022 | 0.020 | 2.8E-01 | -0.025 | 0.020 | 2.1E-01 | 7446 |
| rs12885454 | 14 | 28806589 | PRKD1 | C | A | 0.64 | 0.018 | 0.017 | 2.9E-01 | 0.002 | 0.017 | 8.9E-01 | 7446 |
| rs6091540 | 20 | 50521269 | ZFP64 | C | T | 0.74 | 0.019 | 0.019 | 3.0E-01 | -0.017 | 0.019 | 3.5E-01 | 7446 |
| rs1441264 | 13 | 78478920 | MIR548A2 | A | G | 0.61 | 0.017 | 0.017 | 3.1E-01 | -0.002 | 0.017 | 9.3E-01 | 7446 |
| rs10968576 | 9 | 28404339 | LINGO2 | G | A | 0.31 | 0.017 | 0.017 | 3.4E-01 | -0.007 | 0.017 | 7.0E-01 | 7446 |
| rs17094222 | 10 | 102385430 | HIF1AN | C | T | 0.19 | 0.019 | 0.020 | 3.4E-01 | -0.026 | 0.020 | 2.0E-01 | 7446 |
| rs12016871 | 13 | 26915782 | MTIF3 | T | C | 0.21 | -0.018 | 0.020 | 3.5E-01 | 0.017 | 0.020 | 4.0E-01 | 7446 |
| rs1928295 | 9 | 119418304 | TLR4 | T | C | 0.54 | 0.015 | 0.016 | 3.6E-01 | -0.025 | 0.016 | 1.2E-01 | 7446 |
| rs7899106 | 10 | 87400884 | GRID1 | G | A | 0.05 | 0.034 | 0.039 | 3.8E-01 | -0.040 | 0.038 | 2.9E-01 | 7446 |
| rs16907751 | 8 | 81538012 | ZBTB10 | C | T | 0.94 | 0.030 | 0.036 | 4.1E-01 | 0.024 | 0.035 | 5.0E-01 | 7446 |
| rs9925964 | 16 | 31037396 | KAT8 | A | G | 0.62 | 0.014 | 0.017 | 4.1E-01 | 0.006 | 0.017 | 7.1E-01 | 7446 |
| rs3849570 | 3 | 81874802 | GBE1 | A | C | 0.36 | 0.013 | 0.017 | 4.2E-01 | -0.013 | 0.017 | 4.2E-01 | 7446 |
| rs10733682 | 9 | 128500735 | LMX1B | A | G | 0.48 | 0.012 | 0.016 | 4.6E-01 | -0.023 | 0.016 | 1.5E-01 | 7446 |
| rs2033732 | 8 | 85242264 | RALYL | C | T | 0.74 | 0.013 | 0.019 | 5.0E-01 | 0.014 | 0.018 | 4.5E-01 | 7446 |
| rs11165643 | 1 | 96696685 | PTBP2 | T | C | 0.57 | 0.010 | 0.016 | 5.4E-01 | 0.007 | 0.016 | 6.8E-01 | 7446 |
| rs1016287 | 2 | 59159129 | FLJ30838 | T | C | 0.28 | 0.011 | 0.018 | 5.5E-01 | -0.010 | 0.018 | 5.9E-01 | 7446 |
| rs9914578 | 17 | 1951886 | SMG6 | G | C | 0.21 | 0.011 | 0.020 | 5.6E-01 | 0.005 | 0.020 | 7.9E-01 | 7446 |
| rs3817334 | 11 | 47607569 | MTCH2 | T | C | 0.40 | 0.009 | 0.016 | 5.7E-01 | -0.003 | 0.016 | 8.7E-01 | 7446 |
| rs9540493 | 13 | 65103705 | MIR548X2 | A | G | 0.44 | 0.009 | 0.016 | 5.8E-01 | -0.008 | 0.016 | 6.2E-01 | 7446 |
| rs11057405 | 12 | 121347850 | CLIP1 | G | A | 0.90 | 0.015 | 0.028 | 5.9E-01 | -0.023 | 0.028 | 4.1E-01 | 7446 |
| rs2836754 | 21 | 39213610 | ETS2 | C | T | 0.63 | -0.008 | 0.017 | 6.3E-01 | 0.021 | 0.017 | 2.1E-01 | 7446 |
| rs977747 | 1 | 47457264 | TAL1 | T | G | 0.37 | -0.008 | 0.017 | 6.4E-01 | -0.013 | 0.017 | 4.3E-01 | 7446 |
| rs2075650 | 19 | 50087459 | TOMM40 | A | G | 0.84 | 0.011 | 0.024 | 6.5E-01 | 0.003 | 0.024 | 9.1E-01 | 7446 |
| rs2650492 | 16 | 28240912 | SBK1 | A | G | 0.34 | -0.008 | 0.018 | 6.7E-01 | 0.000 | 0.018 | 9.9E-01 | 7446 |
| rs1460676 | 2 | 164275935 | FIGN | C | T | 0.17 | 0.009 | 0.022 | 6.7E-01 | -0.028 | 0.021 | 1.9E-01 | 7446 |
| rs12429545 | 13 | 53000207 | OLFM4 | A | G | 0.13 | 0.010 | 0.024 | 6.9E-01 | -0.003 | 0.024 | 8.9E-01 | 7446 |
| rs1516725 | 3 | 187306698 | ETV5 | C | T | 0.88 | 0.009 | 0.025 | 7.1E-01 | 0.042 | 0.025 | 9.6E-02 | 7446 |
| rs13201877 | 6 | 137717234 | IFNGR1 | G | A | 0.15 | 0.009 | 0.023 | 7.1E-01 | 0.019 | 0.023 | 4.2E-01 | 7446 |
| rs2176598 | 11 | 43820854 | HSD17B12 | T | C | 0.24 | 0.006 | 0.019 | 7.6E-01 | 0.016 | 0.019 | 3.8E-01 | 7446 |
| rs7239883 | 18 | 38401669 | LOC284260 | G | A | 0.39 | 0.005 | 0.017 | 7.6E-01 | -0.015 | 0.017 | 3.6E-01 | 7446 |
| rs6804842 | 3 | 25081441 | RARB | G | A | 0.58 | 0.004 | 0.016 | 8.0E-01 | 0.014 | 0.016 | 3.9E-01 | 7446 |
| rs3736485 | 15 | 49535902 | DMXL2 | A | G | 0.43 | -0.004 | 0.016 | 8.0E-01 | 0.006 | 0.016 | 7.2E-01 | 7446 |
| rs657452 | 1 | 49362434 | AGBL4 | A | G | 0.39 | 0.004 | 0.017 | 8.0E-01 | -0.031 | 0.017 | 6.1E-02 | 7446 |
| rs12446632 | 16 | 19842890 | GPRC5B | G | A | 0.87 | -0.004 | 0.024 | 8.7E-01 | -0.024 | 0.024 | 3.1E-01 | 7446 |
| rs11688816 | 2 | 62906552 | EHBP1 | G | A | 0.56 | 0.002 | 0.016 | 8.8E-01 | -0.010 | 0.016 | 5.5E-01 | 7446 |
| rs1528435 | 2 | 181259207 | UBE2E3 | T | C | 0.64 | -0.002 | 0.017 | 8.9E-01 | 0.015 | 0.017 | 3.6E-01 | 7446 |
| rs1000940 | 17 | 5223976 | RABEP1 | G | A | 0.28 | 0.002 | 0.018 | 8.9E-01 | -0.005 | 0.018 | 8.0E-01 | 7446 |
| rs11583200 | 1 | 50332407 | ELAVL4 | C | T | 0.39 | 0.002 | 0.016 | 9.2E-01 | -0.003 | 0.016 | 8.4E-01 | 7446 |
| rs7141420 | 14 | 78969207 | NRXN3 | T | C | 0.51 | -0.001 | 0.016 | 9.3E-01 | -0.005 | 0.016 | 7.5E-01 | 7446 |
| rs2176040 | 2 | 226801046 | LOC646736 | A | G | 0.38 | 0.001 | 0.017 | 9.3E-01 | 0.046 | 0.017 | 5.3E-03 | 7446 |
| rs6465468 | 7 | 95007450 | ASB4 | T | G | 0.32 | -0.001 | 0.018 | 9.4E-01 | -0.031 | 0.018 | 8.0E-02 | 7446 |
| rs7243357 | 18 | 55034299 | GRP | T | G | 0.84 | -0.002 | 0.022 | 9.4E-01 | 0.002 | 0.022 | 9.5E-01 | 7446 |
| rs12286929 | 11 | 114527614 | CADM1 | G | A | 0.54 | 0.001 | 0.016 | 9.5E-01 | -0.004 | 0.016 | 7.9E-01 | 7446 |
| rs4256980 | 11 | 8630515 | TRIM66 | G | C | 0.65 | -0.001 | 0.017 | 9.6E-01 | -0.014 | 0.017 | 4.1E-01 | 7446 |
| rs17724992 | 19 | 18315825 | PGPEP1 | A | G | 0.76 | 0.001 | 0.019 | 9.8E-01 | -0.015 | 0.019 | 4.2E-01 | 7446 |
| rs9374842 | 6 | 120227364 | LOC285762 | T | C | 0.74 | 0.000 | 0.018 | 9.8E-01 | -0.011 | 0.018 | 5.6E-01 | 7446 |
| rs11191560 | 10 | 104859028 | NT5C2 | C | T | 0.10 | 0.000 | 0.027 | 9.9E-01 | 0.014 | 0.026 | 5.9E-01 | 7446 |
| rs17405819 | 8 | 76969139 | HNF4G | T | C | 0.70 | 0.000 | 0.018 | 9.9E-01 | 0.017 | 0.017 | 3.3E-01 | 7446 |
|  |  |  |  |  |  |  |  |  |  |  |  |  |  |
| Association for individual BMI SNPs with z-scored ln-transformed BMI and z-scored serum testosterone (T). Linear regression models were adjusted for age, smoking, site and time of day for blood samples, when applicable. Beta and se are expressed in standard deviations of outcome per allele. P-values in bold are statistically significant after Bonferroni adjustment (p<5.2*10^-4^). | | | | | | | | | | | | | |
