# Supplementary material for: Causal relationship between obesity and serum testosterone status in men: A bi-directional mendelian randomization analysis
Source: PLoS One. 2017 Apr 27;12(4):e0176277. doi: 10.1371/journal.pone.0176277 (PMC5407807; doi:10.1371/journal.pone.0176277)
Supplement: S3 Table — (DOCX) [file pone.0176277.s006.docx]

| **S3 Table. Summary of the IV analyses using the weighted and un-weighted genetic risk scores for testosterone.** | | | | | | | | | |  |  |  |  |
| --- | --- | --- | --- | --- | --- | --- | --- | --- | --- | --- | --- | --- | --- |
|  |  | | | | |  |  |  |  |  |  |  |  |
|  |  |  | **1st Stage: GRS vs T** | | | | **2nd stage: GRS vs BMI** | | | | **IV** | | |
| Genetic risk score | Cohort | F | BETA | SE | p | R^2^ | BETA | SE | p | R^2^ | IV | SE | p |
|  | GOOD (n=929) | 21 | -0.26 | 0.06 | 4.0E-06 | 2.3% | -0.06 | 0.06 | 2.6E-01 | 0.1% | 0.24 | 0.22 | 2.8E-01 |
|  | MrOS Sweden (n=1682) | 38 | -0.25 | 0.04 | 5.0E-10 | 2.2% | 0.03 | 0.04 | 4.8E-01 | 0.0% | -0.11 | 0.16 | 4.8E-01 |
|  | SHIP (n=1912) | 53 | -0.30 | 0.04 | 7.1E-14 | 2.7% | -0.02 | 0.04 | 5.5E-01 | 0.0% | 0.08 | 0.13 | 5.5E-01 |
| **_w_GRS_T_** | SHIP Trend (n=427) | 3 | -0.16 | 0.10 | 9.6E-02 | 0.6% | -0.09 | 0.10 | 3.6E-01 | 0.2% | 0.54 | 0.68 | 4.2E-01 |
|  | INTER99 (n=2496) | 76 | -0.31 | 0.03 | 3.4E-19 | 3.0% | -0.03 | 0.04 | 4.1E-01 | 0.0% | 0.09 | 0.12 | 4.2E-01 |
|  |  |  |  |  |  |  |  |  |  |  |  |  |  |
|  | **Meta-analysed combined (n=7446)** | **188** | **-0.28** | **0.02** | **4.7E-45** | **2.5%** | **-0.02** | **0.02** | **3.2E-01** | **0.0%** | **0.07** | **0.32** | **2.1E-01** |
|  |  |  |  |  |  |  |  |  |  |  |  |  |  |
|  | **Pooled combined (n=7446)** | **187** | **-0.28** | **0.02** | **1.5E-44** | **2.4%** | **-0.02** | **0.02** | **2.7E-01** | **0.0%** | **0.07** | **0.07** | **2.7E-01** |
|  |  |  |  |  |  |  |  |  |  |  |  |  |  |
|  |  |  |  |  |  |  |  |  |  |  |  |  |  |
| **_uw_GRS_T_** | GOOD (n=929) | 10 | -0.14 | 0.04 | 1.6E-03 | 1.1% | -0.04 | 0.04 | 3.3E-01 | 0.1% | 0.31 | 0.33 | 3.5E-01 |
|  | MROS (n=1682) | 37 | -0.20 | 0.03 | 6.5E-10 | 2.2% | 0.02 | 0.03 | 5.2E-01 | 0.0% | -0.11 | 0.16 | 5.2E-01 |
|  | SHIP (n=1912) | 43 | -0.20 | 0.03 | 1.8E-11 | 2.2% | 0.00 | 0.03 | 9.4E-01 | 0.0% | -0.01 | 0.15 | 9.4E-01 |
|  | SHIP Trend (n=427) | 1 | -0.08 | 0.07 | 2.4E-01 | 0.3% | -0.03 | 0.06 | 6.6E-01 | 0.0% | 0.37 | 0.90 | 6.8E-01 |
|  | INTER99 (n=2496) | 71 | -0.21 | 0.02 | 4.1E-18 | 2.8% | -0.01 | 0.03 | 7.4E-01 | 0.0% | 0.04 | 0.12 | 7.4E-01 |
|  |  |  |  |  |  |  |  |  |  |  |  |  |  |
|  | **Meta-analysed combined (n=7446)** | **156** | **-0.19** | **0.01** | **3.9E-38** | **2.1%** | **0.00** | **0.02** | **7.8E-01** | **0.0%** | **0.02** | **0.08** | **7.8E-01** |
|  |  |  |  |  |  |  |  |  |  |  |  |  |  |
|  | **Pooled combined (n=7446)** | **157** | **-0.19** | **0.01** | **7.6E-38** | **2.1%** | **-0.01** | **0.01** | **7.1E-01** | **0.0%** | **0.03** | **0.07** | **7.1E-01** |
|  |  |  |  |  |  |  |  |  |  |  |  |  |  |
| IV is the instrumental variable ratio which was calculated as the ratio between the association of the weighted (_w_GRS_T_) and un-weighted (_uw_GRS_T_) genetic risk score for serum testosterone with z-scored ln-transformed BMI and the association between the genetic risk scores and z-scored serum testosterone. Linear regression models were adjusted for age, smoking, site and time of day for blood samples, when applicable. Beta and se are expressed in standard deviations per unit of _w_GRS_T_ and _w_GRS_T_. F is the F statistics. R^2^ is the variance explained. T = serum testosterone. Both fixed effect meta-analysis (Meta-analysed combined) and analyses with pooled data (Pooled combined) were used, yielding similar results. | | | | | | | | | | | | | |
