# Supplementary material for: Causal relationship between obesity and serum testosterone status in men: A bi-directional mendelian randomization analysis
Source: PLoS One. 2017 Apr 27;12(4):e0176277. doi: 10.1371/journal.pone.0176277 (PMC5407807; doi:10.1371/journal.pone.0176277)
Supplement: S4 Table — (DOCX) [file pone.0176277.s007.docx]

| **S4 Table. Summary of the association for the three testosterone-associated SNPs with testosterone and BMI.** | | | | | | | | | | | | |  |
| --- | --- | --- | --- | --- | --- | --- | --- | --- | --- | --- | --- | --- | --- |
|  |  |  |  |  |  |  |  |  |  |  |  |  |  |
|  |  | Position | Nearest | Effect | Other | Effect Allele | **Testosterone** | | | **BMI** | | |  |
| SNP | Chr | (bp) | Gene | Allele | Allele | Frequency | Beta_T_ | SE_T_ | P value_T_ | Beta_BMI_ | SE_BMI_ | P value_BMI_ | N |
|  |  |  |  |  |  |  |  |  |  |  |  |  |  |
| rs12150660 | 17 | 7462640 | SHBG | G | T | 0.76 | -0.23 | 0.02 | 7.8E-32 | -0.01 | 0.02 | 5.3E-01 | 7446 |
| rs6258 | 17 | 7475403 | SHBG | T | C | 0.02 | -0.53 | 0.07 | 7.1E-15 | -0.09 | 0.07 | 1.7E-01 | 7446 |
| rs5934505 | X | 8873826 | FAM9B | T | C | 0.75 | -0.09 | 0.03 | 4.7E-04 | 0.02 | 0.03 | 3.7E-01 | 7446 |
|  |  |  |  |  |  |  |  |  |  |  |  |  |  |
| Association for individual testosterone SNPs with z-scored serum testosterone (T) and z-scored ln-transformed BMI. Linear regression models were adjusted for age, smoking, site and time of day for blood samples, when applicable. Beta and se are expressed in standard deviations of outcome per allele. | | | | | | | | | | | | |  |
