# Supplementary material for: Causal relationship between obesity and serum testosterone status in men: A bi-directional mendelian randomization analysis
Source: PLoS One. 2017 Apr 27;12(4):e0176277. doi: 10.1371/journal.pone.0176277 (PMC5407807; doi:10.1371/journal.pone.0176277)
Supplement: S5 Table — (DOCX) [file pone.0176277.s008.docx]

| **S5 Table. Summary of the IV analyses using independent genetic risk scores for BMI** | | | | | | |  |  |  |  |  |
| --- | --- | --- | --- | --- | --- | --- | --- | --- | --- | --- | --- |
|  | | | |  |  |  |  |  |  |  |  |
|  | **1st Stage: GRS vs BMI** | | | | **2nd stage: GRS vs T** | | | | **IV** | | |
| Genetic risk score / SNP | BETA | SE | p | R^2^ | BETA | SE | p | R^2^ | IV | SE | p |
|  |  |  |  |  |  |  |  |  |  |  |  |
| **_w_GRS_BMI - 96 SNPs_** | **0.02** | **0.00** | **8.5E-29** | **1.3%** | **-0.01** | **0.00** | **8.9E-03** | **0.9%** | **-0.26** | **0.10** | **1.1E-02** |
| **_uw_GRS_BMI - 96 SNPs_** | **0.02** | **0.00** | **2.8E-24** | **1.1%** | **-0.01** | **0.00** | **2.7E-03** | **0.1%** | **-0.32** | **0.11** | **4.0E-03** |
| **FTO** | **0.07** | **0.01** | **4.2E-07** | **0.3%** | **-0.03** | **0.02** | **4.7E-02** | **0.0%** | **-0.43** | **0.23** | **6.5E-02** |
|  |  |  |  |  |  |  |  |  |  |  |  |
| IV is the instrumental variable ratio which was calculated as the ratio between the association of the genetic risk score (GRS) with z-scored ln-transformed BMI and the association between the GRS and z-scored serum testosterone (T). Linear regression models were adjusted for age, smoking, site and time of day for blood samples, when applicable. Beta and se are expressed in standard deviations per unit of risk score. _w_GRS_BMI - 96 SNPs_ = Weighted genetic risk score based on SNPs associated with BMI excluding the SNP located within the FTO gene. _uw_GRS_BMI - 96 SNPs_ = Un-weighted genetic risk score based on SNPs associated with BMI excluding the SNP located within the FTO gene. FTO = SNP located within the FTO gene. F is the F statistic. R2 is the variance explained. | | | | | | | | | | | |
