# Supplementary material for: Causal relationship between obesity and serum testosterone status in men: A bi-directional mendelian randomization analysis
Source: PLoS One. 2017 Apr 27;12(4):e0176277. doi: 10.1371/journal.pone.0176277 (PMC5407807; doi:10.1371/journal.pone.0176277)
Supplement: S6 Table — (DOCX) [file pone.0176277.s009.docx]

| **S6 Table. Associations between testosterone SNPs and BMI when evaluated in the Giant consortium.** | | |
| --- | --- | --- |
|  | | |
| **SNPs/Risk score** | **Per allele Change in BMI, kg/m^2^ (95% CI)** | **P value** |
|  |  |  |
| rs6258 | 0.000 (-0.038 - 0.039) | 0.98 |
| rs12150660 | -0.011 (-0.023 - 0.001) | 0.07 |
| GRS_T_ | -0.012 (-0.029 - 0.005)* | 0.17 |
|  |  |  |
| The GIANT consortium included up to 104,349 men of European ancestry (Locke et al, Nature, 2015). The association for GRS_T_ (including SNPs rs6258 and rs12150660) was calculated as described in Ehret et al (9). * per allele change in BMI. | | |
|  |  |  |
