# Supplementary material for: Causal relationship between obesity and serum testosterone status in men: A bi-directional mendelian randomization analysis
Source: PLoS One. 2017 Apr 27;12(4):e0176277. doi: 10.1371/journal.pone.0176277 (PMC5407807; doi:10.1371/journal.pone.0176277)
Supplement: S7 Table — (DOCX) [file pone.0176277.s010.docx]

| **S7 Table. Summary of the IV analyses using genetic risk scores for testosterone.** | | | | |  |  |  |  |  |  |  |  |  |
| --- | --- | --- | --- | --- | --- | --- | --- | --- | --- | --- | --- | --- | --- |
|  | | | | |  |  |  |  |  |  |  |  |  |
|  |  | **1st Stage: GRS vs Testosterone** | | | | | **2nd stage: GRS vs BMI** | | | | **IV** | | |
| Genetic risk score | Cohort | F | BETA | SE | p | R^2^ | BETA | SE | p | R^2^ | IV | SE | p |
|  |  |  |  |  |  |  |  |  |  |  |  |  |  |
| **_w_GRS_T - Within SHBG_** | GOOD (n=929) | 19 | -0.24 | 0.05 | 1.2E-05 | 2.0% | -0.07 | 0.06 | 2.3E-01 | 0.2% | 0.27 | 0.24 | 2.5E-01 |
|  | MrOS Sweden (n=1682) | 40 | -0.24 | 0.04 | 2.0E-10 | 2.3% | 0.01 | 0.04 | 7.9E-01 | 0.0% | -0.04 | 0.16 | 7.9E-01 |
|  | SHIP (n=1912) | 38 | -0.23 | 0.04 | 3.1E-10 | 1.9% | -0.01 | 0.04 | 7.9E-01 | 0.0% | 0.04 | 0.16 | 7.9E-01 |
|  | SHIP Trend (n=427) | 3 | -0.14 | 0.08 | 7.0E-02 | 0.7% | -0.03 | 0.08 | 6.7E-01 | 0.0% | 0.23 | 0.56 | 6.8E-01 |
|  | INTER99 (n=2496) | 69 | -0.26 | 0.03 | 1.6E-17 | 2.7% | -0.02 | 0.03 | 4.4E-01 | 0.0% | 0.09 | 0.12 | 4.5E-01 |
|  |  |  |  |  |  |  |  |  |  |  |  |  |  |
|  | **Meta-analysed combined (n=7446)** | **166** | **-0.24** | **0.02** | **4.9E-40** | **2.2%** | **-0.02** | **0.02** | **3.4E-01** | **0.0%** | **0.07** | **0.08** | **3.4E-01** |
|  |  |  |  |  |  |  |  |  |  |  |  |  |  |
|  | **Pooled combined (n=7446)** | **164** | **-0.24** | **0.02** | **1.9E-39** | **2.2%** | **-0.02** | **0.02** | **2.7E-01** | **0.0%** | **0.08** | **0.07** | **2.7E-01** |
|  |  |  |  |  |  |  |  |  |  |  |  |  |  |
| **_w_GRS_T - Not within SHBG_** | GOOD (n=929) | 0 | 0.04 | 0.07 | 5.6E-01 | 0.0% | 0.00 | 0.07 | 9.8E-01 | 0.0% | -0.05 | 1.72 | 9.8E-01 |
|  | MrOS Sweden (n=1682) | 2 | -0.09 | 0.06 | 1.1E-01 | 0.1% | 0.04 | 0.06 | 4.4E-01 | 0.0% | -0.49 | 0.70 | 4.8E-01 |
|  | SHIP (n=1912) | 7 | -0.14 | 0.05 | 8.3E-03 | 0.3% | 0.03 | 0.05 | 5.9E-01 | 0.0% | -0.20 | 0.38 | 6.0E-01 |
|  | SHIP Trend (n=427) | 0 | 0.06 | 0.11 | 6.0E-01 | 0.1% | -0.01 | 0.11 | 8.9E-01 | 0.0% | -0.26 | 1.97 | 8.9E-01 |
|  | INTER99 (n=2496) | 8 | -0.13 | 0.04 | 2.8E-03 | 0.3% | 0.02 | 0.05 | 6.1E-01 | 0.0% | -0.18 | 0.35 | 6.1E-01 |
|  |  |  |  |  |  |  |  |  |  |  |  |  |  |
|  | **Meta-analysed combined (n=7446)** | **16** | **-0.09** | **0.03** | **4.7E-04** | **0.2%** | **0.02** | **0.03** | **3.7E-01** | **0.0%** | **-0.26** | **0.30** | **3.9E-01** |
|  |  |  |  |  |  |  |  |  |  |  |  |  |  |
|  | **Pooled combined (n=7446)** | **12** | **-0.09** | **0.03** | **3.3E-04** | **0.2%** | **0.02** | **0.02** | **3.5E-01** | **0.0%** | **-0.24** | **0.26** | **3.6E-01** |
|  |  |  |  |  |  |  |  |  |  |  |  |  |  |
| IV is the instrumental variable ratio which was calculated as the ratio between the association of the genetic risk score (GRS) with z-scored ln-transformed BMI and the association between the GRS and z-scored serum testosterone (T). Linear regression models were adjusted for age, smoking, site and time of day for blood samples, when applicable. Beta and se are expressed in standard deviations per weighted risk allele.  _w_GRS_T - Within SHBG_ = Weighted genetic risk score on SNPs (located within the SHBG gene) associated with T.  _w_GRS_T - Not within SHBG_ = Weighted genetic risk score on SNP (not located within the SHBG gene) associated with T. F is the F statistics. R^2^ is the variance explained. | | | | | | | | | | | | | |
