# Supplementary material for: Causal relationship between obesity and serum testosterone status in men: A bi-directional mendelian randomization analysis
Source: PLoS One. 2017 Apr 27;12(4):e0176277. doi: 10.1371/journal.pone.0176277 (PMC5407807; doi:10.1371/journal.pone.0176277)
Supplement: S1 File — (DOCX) [file pone.0176277.s011.docx]

# Supplemental methods

**Study specific cohort information**

**Gothenburg Osteoporosis and Obesity Determinants (GOOD) study**

The Gothenburg Osteoporosis and Obesity Determinants (GOOD) study was initiated to determine both environmental and genetic factors involved in the regulation of bone and fat mass [1]. Male study subjects were randomly identified in the greater Gothenburg area in Sweden using national population registers, contacted by telephone, and invited to participate. To be enrolled in the GOOD study, subjects had to be between 18 and 20 years of age. There were no other exclusion criteria, and 49% of the study candidates agreed to participate (n = 1068). Exclusion criteria in the present study included chemical or surgical castration and/or medications affecting sex hormones such as steroid 5-alpha reductase inhibitors, and sex hormone antagonists. Only subjects with full information on both genotypes and phenotypes (n=929) were included in the present study. The study was approved by the ethics committee at the University of Gothenburg. Written and oral informed consent was obtained from all study participants.

**MROS Sweden**

The MrOS study is a prospective multicenter study including elderly men from Hong Kong, the United States, and Sweden [2]. The present study includes the Gothenburg (n= 1010) and Malmö (n=1005) part of the Swedish cohort. At baseline, letters were sent to a randomly selected group of subjects (men aged 69 to 81 years old) identiﬁed in national population registers and contacted by telephone. To be eligible for the study, the participants had to be able to walk without aid, sign an informed consent, and complete a questionnaire. The inclusion rate at baseline for the Swedish part of the MrOS study was 45% [3].

In the present study, exclusion criteria included chemical or surgical castration and/or medications affecting sex hormones such as steroid 5-alpha reductase inhibitors, and sex hormone antagonists. Only subjects with full information on both genotypes and phenotypes were included (n=1682) in the present study. The study was approved by the ethics committee at the University of Gothenburg and Lund. Written and oral informed consent was obtained from all study participants.

**Study of Health in Pomerania (SHIP)** The Study of Health in Pomerania (SHIP) is a longitudinal population-based cohort study in West Pomerania, a region in the northeast of Germany, assessing the prevalence and incidence of common population-relevant diseases and their risk factors [4].

From the entire study population of 212157 inhabitants living in the area, a sample was selected from the population registration offices, where all German inhabitants are registered. Only individuals with German citizenship and main residency in the study area were included. A two-stage cluster sampling method was adopted from the WHO MONICA Project Augsburg, Germany. In a first step, the three cities of the region (with 17076 to 65977 inhabitants) and the 12 towns (with 1516 to 3044 inhabitants) were selected. Further 17 out of 97 smaller towns (with less than 1500 inhabitants) were drawn at random. In a second step, from each of the selected communities, subjects were drawn at random, proportional to the population size of each community and stratified by age and gender. Finally, 7008 subjects aged 20 to 79 years were sampled, with 292 persons of each gender in each of the twelve five-year age strata. In order to minimize drop-outs by migration or death, subjects were selected in two waves. The net sample (without migrated or deceased persons) comprised 6267 eligible subjects. Selected persons received a maximum of three written invitations. In case of non-response, letters were followed by a phone call or by home visits if contact by phone was not possible. The SHIP population finally comprised 4308 participants at baseline (corresponding to a final response of 68.8%).

Of the 2116 male participants in this study, men with missing genotype or phenotype data were excluded. Exclusion criteria in the present study included chemical or surgical castration and/or medications affecting sex hormones such as steroid 5-alpha reductase inhibitors, and sex hormone antagonists. Altogether, for the present study, valid data were available in 1912 males of the SHIP baseline.

**SHIP-Trend**

Baseline examinations for SHIP-Trend were carried out between 2008 and 2012. Of a stratified random net sample of 8826 individuals aged 20-79 years with German citizenship and main residency in the study area, 4420 individuals (2145 men) finally participated (response 50.1%). The present project is based on subjects aged 20 to 81 years of the SHIP-Trend study population. Exclusion criteria in the present study included chemical or surgical castration and/or medications affecting sex hormones such as steroid 5-alpha reductase inhibitors, and sex hormone antagonists. Altogether, valid data with genotype and phenotype data were available in 427 males of the SHIP Trend cohort.

These studies were approved by the ethics committee at the University of Greifswald. Written and oral informed consent was obtained from all study participants.

**Inter99**

The Inter99 study carried out in 1999-2001 included invitation of 12934 persons aged 30-60 years drawn from an age- and sex-stratified random sample of the general population of the Western part of Copenhagen [5]. The participation rate was 52.5%, and the study included 6784 persons. The Inter99 study was a population-based randomized controlled trial (CT00289237, ClinicalTrials.gov) and investigated the effects of lifestyle intervention on CVD. The health examination included a self-administered questionnaire, various blood tests and a physical examination that formed the basis of a risk assessment. In the present study only male subjects with full information about both genotypes and phenotypes were included (n=2496). The study was approved by the ethics committee at the University of Copenhagen. Written and oral informed consent was obtained from all study participants.

**Information on genotyping and imputation**

**GOOD**

Genotyping was performed using the Illumina HumanHap610 Quad arrays and genotypes were called using the BeadStudio calling algorithm. Genotypes from 938 individuals passed the sample quality control criteria [exclusion criteria: sample call rate < 97.5%, gender discrepancy with genetic data from X-linked markers, excess autosomal heterozygosity > 0.33 ~ FDR < 0.1%, duplicates and/or first degree relatives identified using IBS probabilities (> 97%), ethnic outliers (3 SD away from the population mean) using multi-dimensional scaling analysis with four principal components]. Across 22 duplicate samples, genotype concordance exceeded 99.9%. Genotypes were imputed for all polymorphic SNPs (521,160 with MAF ≥ 1%, SNP call rate ≥ 98% and HWE p value ≥ 10-6) using the MACH software, based upon phased autosomal chromosomes of the HapMap CEU Phase II panel (release 22, build 36), orientated on the positive strand. Uncertainty in genotype prediction was accounted for by utilizing the dosage information from MACH.

**MROS Sweden**

*Gothenburg part:* Genotyping, imputation and quality controls were performed using the Illumina HumanOmni1_Quad_v1-0 B array. Genotypes were called using the Illumina’s BeadStudio calling algorithm. The sample quality control exclusion criteria were sample call rate < 97%, excessive autosomal heterozygozity, first and second degree relatives, genotypic sex mismatch using X and Y chromosome probe intensities and gross chromosome abnormalities. Genotyped SNPs with GenTrain scores <0.6, cluster separation scores <0.4, call rates <97%, or MAF <0.01 were excluded. Also, autosomal SNPs with Hardy-Weinberg Equilibrium [HWE] P-value <10^-4^ were excluded and genotype clusters for SNPs on chrX, chrY, chrXY and chrMT were reviewed manually. 714543 autosomal SNPs passed quality control. Imputation was done using MaCH (v 1.0.17,phasing) [6] and Minimac v 2011-08-12 beta [7] for HapMap phase II release 22 build 36, oriented on the positive strand.

*Malmö part:* Genotyping, imputation and quality controls were performed using the HumanOmniExpress-12v1_B build 36. The sample quality control exclusion criteria were sample call rate < 97.5%, missing data, excessive autosomal heterozygozity, familiar relationship (one sample excluded), genotypic sex mismatch, non-caucasians and gross chromosome abnormalities. SNPs with call rates<95% were excluded. 725409 autosomal SNPs passed quality control. Imputation was done using a two steps procedure using Minmac and MACH for HapMap phase II release 22 build 36 (oriented on the positive strand) for the phasing and imputation respectively. CEU HapMap samples were used as a reference panel for the consensus phased haplotypes. 927 subjects were available with quality controlled imputed data.

The number of SNPs imputed was 2543887, which is the expected number after using the HapMap Phase II CEU reference panel. The mean MACH r-squared for the whole data was of 0.95, median (1.00), sd (0.14).MAF > 1% and r2 > 0.3 after the imputation process is: 2447978.

**SHIP**

The SHIP samples were genotyped using the Affymetrix Human SNP Array 6.0. Hybridisation of genomic DNA was done in accordance with the manufacturer’s standard recommendations. The genetic data analysis workflow was created using the Software InforSense. Genotypes were determined using the Birdseed2 clustering algorithm. For quality control purposes, several control samples where added. On the chip level, only subjects with a genotyping rate on QC probesets (QC callrate) of at least 86% were included. All remaining arrays had a sample callrate > 92%. The overall genotyping efficiency of the GWA was 98.55 %. Imputation of genotypes in SHIP was performed with the software IMPUTE v0.5.0 based on HapMap phase II release 22 build 36. Genome-wide association tests were performed using QUICKTEST v0.95 (http://toby.freeshell.org/software/quicktest.shtml). Uncertainties for imputed genotypes were taken into account for association testing.

**SHIP-Trend**

Genotyping of the SHIP-Trend subjects was performed using the Illumina HumanOmni2.5-Quad. DNA from whole blood was prepared using the Gentra Puregene Blood Kit (Qiagen, Hilden, Germany) according to the manufacturer's protocol. Subsequent sample processing and array hybridization were performed as described by the manufacturer (Illumina). The final sample call rate was 99.51%. Imputation of genotypes in the SHIP-Trend cohort was performed with the software IMPUTE v2.1.2.3 against the HapMap II (CEU v22, Build 36) reference panel. The total number of SNPs after imputation and quality control was 3437411.

**Inter99**

In Inter99 all SNPs besides three (rs6258, rs5934505 and rs12150660) were genotyped by the Illumina Cardio-Metabo BeadChip. Genotypes were called using GenomeStudio software (version 2011.1, Illumina). Individuals with 1st or 2nd degree familial relationship, extreme inbreeding coefficient, low call rate, mislabeled sex and high discordance to previous genotypings were excluded. Genotyping quality for each variant was assessed by the call-rate (>95%) and presence of Hardy-Weinberg equilibrium (P>0.01). The remaining three SNPs, rs6258, rs5934505 and rs12150660, were genotyped KASPar SNP Genotyping system (KBioscience, Hoddesdon, UK). Success-rates of the genotyping were above 98% for all 3 SNPs. Error rates were 0% for all 3 variants, as estimated from re-genotyping of minimum 348 duplicate samples.

**Development of genetic risk scores for BMI and serum testosterone**

The 97 BMI-associated SNPs identified in a recent large-scale GWAS on BMI were used to construct a weighted genetic risk score, _w_GRS_BMI_. Whenever a SNP was not available, a proxy SNP was used (with r2>0.8) instead. In the Inter99 cohort, which used directly genotyped SNPs, a proxy SNP was not available for one of the missing SNPs resulting in 96, rather than 97, SNPs being used for the GRS_BMI_s in this cohort. The _w_GRS_BMI_, was calculated for each individual by summing the number of risk alleles (dosage) for each SNP weighted by the SNP’s estimated effect size of its BMI-increasing allele in men [8]. The weights were then transformed to have a mean=1 by dividing each effect size by the mean of the BMI-increasing effect sizes.

The un-weighted genetic risk scores of BMI (_uw_GRS_BMI_) and serum testosterone (_uw_GRS_T_), and respectively, were developed by summing the number of BMI-increasing alleles and T-decreasing alleles, respectively.

**Genetic risk score for serum testosterone (T) based on SNPs located within or outside the SHBG gene**

Due to the complexity of the factors determining T synthesis and bio-availability, we constructed two separate weighted risk scores based on SNPs located in (_w_GRS_T – Within SHBG_: rs6258, rs12150660) and outside the SHBG gene (_w_GRS_T – Not within SHBG_: rs5934505 on the X chromosome).

**Testosterone-associated SNPs in the GIANT consortium**

Due to the fact that SNPs on the X chromosome were not included in the GIANT meta analysis we could not perform this analysis for rs5934505. Rs6258 and rs12150660 were combined into the _w_GRS_T – Within SHBG_ using an approximation method described by Ehret et al [9]. In short, both SNP’s association with BMI was weighted according to its predefined age-adjusted effect size and meta-analyzed using an inverse-variance method with the other SNP.

**Covariates**

**GOOD**

Measurement of BMI: Height was measured using a wall-mounted stadiometer, and weight was measured to the nearest 0.1 kg. Body mass index was calculated as weight (kg)/height (m2).

Measurement of smoking: A standardized questionnaire was used to collect information about smoking.

**MROS Sweden**

Measurement of BMI: Height was measured using a Harpenden stadiometer, and weight was measured by a standard balance beam or an electric scale. Two consecutive measurements of height were performed in the same session, and the average of these measurements was calculated. If there was a difference of ≥5 mm between the first two measurements, a third measurement was performed, and the average of the two values with the least mutual discrepancy was calculated.

Measurement of smoking: A standardized questionnaire was used to collect information about smoking habits.

**SHIP & SHIP-Trend**

Measurement of BMI: Body mass index (BMI) was calculated as weight in kilograms divided by height in square meters measured according to standard protocols using digital scales (Seca 862, Seca Germany) and a measuring stick (Seca 220, Seca, Germany).

Measurement of smoking: A computer-assisted personal interview was used to collect information about smoking.

**Inter99**

Height was measured without shoes. Weight was measured with light clothes and without shoes using a Tanita Body Composition Analyzer TBF-300. The device was calibrated annually. Body mass index (BMI) was calculated as weight (kg) divided by height (m) squared. Information on smoking habits was obtained by a self-completed questionnaire.

**Statistical analysis**

An important assumption in MR analyses is that the instrumental variables, in our case the genetic risk scores do not have an effect on the outcome independently of its effect on the exposure, e.g. pleiotropy. Similar instrumental variable estimates acquired using two independent instruments would provide suggestive evidence against the existence of a pleiotropic effect, as it would be unlikely that both instruments had shared pleiotropy [10, 11]. In order to rule out that the effect of BMI on T was the result of pleiotropy, we created two independent instruments based on the BMI SNPs. The two independent genetic instruments were rs1558902 in FTO (the individual SNP with the largest effect size on BMI identified in the GWAS by Locke et al), and a weighted allelic score constructed from the remaining 96 SNPs associated with BMI, analogous to the approach adopted by Taylor et al [8, 11].

Linearity is another assumption required for the estimation of the size of the causal effect and was assessed by adding a quadratic genetic risk score term to the regression analyses.

The IV ratio for BMI’s effect on serum T was calculated as the meta-analyzed effect of GRS_BMI_ on serum T divided by the meta-analyzed effect of GRS_BMI_ on BMI. An identical approach was used when estimating the effect of serum T on BMI. The variance of the IV ratio was estimated using a Taylor expansion [12].

All meta-analyses used a fixed-effect inverse variance model unless there was evidence of heterogeneity of associations between the studies, in which case a random effects model was used [13].

**Supplemental figure legends**

**S1 Fig. The genetic risk scores used are not associated with age or smoking.** Fixed effect meta-analysis of the associations for A) the weighted genetic risk score on BMI (_w_GRS_BMI_) with age, B) the _w_GRS_BMI_ with smoking, C) the weighted genetic risk score on serum testosterone (T; _w_GRS_T_) with age and D) the _w_GRS_T_ with smoking (n=7446). Linear regression (A, C) and logistic regression (B, D) models were adjusted for site. Effect sizes are given as (A, C) standard deviation (SD) age or (C, D) odds ratio (OR) for smoking per weighted risk allele.

**S2 Fig. Power analysis.** If the genetic association would be of similar magnitude as the observed association, then the present study is adequately powered. We would then have approximately similar power to detect an association between BMI and serum testosterone (T) using the weighted genetic risk score on BMI (_w_GRS_BMI_; 95%) compared with an equal sized effect in the other direction using the weighted genetic risk score on serum T (_w_GRS_T_; 98%). However, a power analysis based on half the observed association between BMI and serum T (½β) indicated that our analyses would not have been adequately powered if this was the case. The red vertical line corresponds to the sample size used in the present study (n=7446).

**S3 Fig. Associations between genetic risk scores and SHBG.** Fixed effect meta-analysis of the associations for A) the weighted genetic risk score on BMI (_w_GRS_BMI_) with serum SHBG and B) the weighted genetic risk score on serum testosterone (T; _w_GRS_T_) with serum SHBG (n=7370). Linear regression models were adjusted for age, smoking, blood sampling time and site. Effect sizes are given as standard deviation (SD) serum SHBG per weighted risk allele.

**References Supplemental Material**

1. Lorentzon M, Swanson C, Andersson N, Mellstrom D, Ohlsson C. Free testosterone is a positive, whereas free estradiol is a negative, predictor of cortical bone size in young Swedish men: the GOOD study. J Bone Miner Res. 2005;20(8):1334-41. doi: 10.1359/JBMR.050404. PubMed PMID: 16007330.

2. Orwoll E, Blank JB, Barrett-Connor E, Cauley J, Cummings S, Ensrud K, et al. Design and baseline characteristics of the osteoporotic fractures in men (MrOS) study--a large observational study of the determinants of fracture in older men. Contemp Clin Trials. 2005;26(5):569-85. doi: 10.1016/j.cct.2005.05.006. PubMed PMID: 16084776.

3. Mellstrom D, Johnell O, Ljunggren O, Eriksson AL, Lorentzon M, Mallmin H, et al. Free testosterone is an independent predictor of BMD and prevalent fractures in elderly men: MrOS Sweden. J Bone Miner Res. 2006;21(4):529-35. doi: 10.1359/jbmr.060110. PubMed PMID: 16598372.

4. Volzke H, Alte D, Schmidt CO, Radke D, Lorbeer R, Friedrich N, et al. Cohort profile: the study of health in Pomerania. International journal of epidemiology. 2011;40(2):294-307. doi: 10.1093/ije/dyp394. PubMed PMID: 20167617.

5. Jorgensen T, Borch-Johnsen K, Thomsen TF, Ibsen H, Glumer C, Pisinger C. A randomized non-pharmacological intervention study for prevention of ischaemic heart disease: baseline results Inter99. Eur J Cardiovasc Prev Rehabil. 2003;10(5):377-86. doi: 10.1097/01.hjr.0000096541.30533.82. PubMed PMID: 14663300.

6. Li YA. Mach 1.0: rapid haplotype reconstruction and missing genotype inference [abstract 2290/C]. Am J Hum Genet. 2006.

7. Howie B, Fuchsberger C, Stephens M, Marchini J, Abecasis GR. Fast and accurate genotype imputation in genome-wide association studies through pre-phasing. Nat Genet. 2012;44(8):955-9. doi: 10.1038/ng.2354. PubMed PMID: 22820512; PubMed Central PMCID: PMCPMC3696580.

8. Locke AE, Kahali B, Berndt SI, Justice AE, Pers TH, Day FR, et al. Genetic studies of body mass index yield new insights for obesity biology. Nature. 2015;518(7538):197-206. doi: 10.1038/nature14177. PubMed PMID: 25673413; PubMed Central PMCID: PMCPMC4382211.

9. Ehret GB, Munroe PB, Rice KM, Bochud M, Johnson AD, Chasman DI, et al. Genetic variants in novel pathways influence blood pressure and cardiovascular disease risk. Nature. 2011;478(7367):103-9. doi: 10.1038/nature10405. PubMed PMID: 21909115; PubMed Central PMCID: PMCPMC3340926.

10. Davey Smith G. Use of genetic markers and gene-diet interactions for interrogating population-level causal influences of diet on health. Genes Nutr. 2011;6(1):27-43. doi: 10.1007/s12263-010-0181-y. PubMed PMID: 21437028; PubMed Central PMCID: PMCPMC3040803.

11. Taylor PN, Richmond R, Davies N, Sayers A, Stevenson K, Woltersdorf W, et al. Paradoxical Relationship Between Body Mass Index and Thyroid Hormone Levels: A Study Using Mendelian Randomization. The Journal of clinical endocrinology and metabolism. 2016;101(2):730-8. doi: 10.1210/jc.2015-3505. PubMed PMID: 26595101.

12. Thomas DC, Lawlor DA, Thompson JR. Re: Estimation of bias in nongenetic observational studies using "Mendelian triangulation" by Bautista et al. Annals of epidemiology. 2007;17(7):511-3. doi: 10.1016/j.annepidem.2006.12.005. PubMed PMID: 17466535.

13. Borenstein M. Introduction to meta-analysis. Chichester, UK: John Wiley & Sons; 2009.
